# Aberrant upregulation of CaSR promotes pathological new bone formation in ankylosing spondylitis

**DOI:** 10.15252/emmm.202012109

**Published:** 2020-12-01

**Authors:** Xiang Li, Siwen Chen, Zaiying Hu, Dongying Chen, Jianru Wang, Zemin Li, Zihao Li, Haowen Cui, Guo Dai, Lei Liu, Haitao Wang, Kuibo Zhang, Zhaomin Zheng, Zhongping Zhan, Hui Liu

**Affiliations:** ^1^ Department of Spine Surgery The First Affiliated Hospital Sun Yat‐sen University Guangzhou China; ^2^ Guangdong Province Key Laboratory of Orthopaedics and Traumatology Guangzhou China; ^3^ Department of Rheumatology and Immunology The Sixth Affiliated Hospital Sun Yat‐sen University Guangzhou China; ^4^ Department of Rheumatology and Immunology The First Affiliated Hospital Sun Yat‐sen University Guangzhou China; ^5^ Department of Spine Surgery The Fifth Affiliated Hospital Sun Yat‐sen University Guangzhou China

**Keywords:** ankylosing spondylitis, CaSR, inflammation, inflammatory cytokines, pathological new bone formation, Musculoskeletal System

## Abstract

Pathological new bone formation is a typical pathological feature in ankylosing spondylitis (AS), and the underlying molecular mechanism remains elusive. Previous studies have shown that the calcium‐sensing receptor (CaSR) is critical for osteogenic differentiation while also being highly involved in many inflammatory diseases. However, whether it plays a role in pathological new bone formation of AS has not been reported. Here, we report the first piece of evidence that expression of CaSR is aberrantly upregulated in entheseal tissues collected from AS patients and animal models with different hypothetical types of pathogenesis. Systemic inhibition of CaSR reduced the incidence of pathological new bone formation and the severity of the ankylosing phenotype in animal models. Activation of PLCγ signalling by CaSR promoted bone formation both *in vitro* and *in vivo*. In addition, various inflammatory cytokines induced upregulation of CaSR through NF‐κB/p65 and JAK/Stat3 pathways in osteoblasts. These novel findings suggest that inflammation‐induced aberrant upregulation of CaSR and activation of CaSR‐PLCγ signalling in osteoblasts act as mediators of inflammation, affecting pathological new bone formation in AS.

## Graphical abstract

This study identifies a critical role of inflammation‐induced aberrant upregulation of CaSR in the process of pathological new bone formation in ankylosing spondylitis (AS). Targeting CaSR may be a novel potential therapeutic strategy to slow down the progression of axial structural ankylosis.

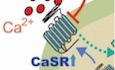

**The paper explained**

**Problem**

Upregulation of CaSR was found in AS spinal samples and animal models. Whether CaSR plays a role in the process of pathological new bone formation in AS is unknown.

**Results**

The expression of CaSR was upregulated in Runx2^+^ and OCN^+^ osteoblasts accumulated in the potential bone‐forming sites in the tissues from AS patients and animal models. Systemic administration of CaSR antagonist NPS‐2143 attenuated pathological new bone formation in animal models. Activation of PLCγsignalling by CaSR promoted osteogenic differentiation and pathological new bone formation. In addition, various inflammatory cytokines including TNFα and interleukin family induced upregulation of CaSR through NF‐κB/p65 and JAK/Stat3 signalling pathways in osteoblasts.

**Impact**

Inflammation‐induced upregulation of CaSR acts as a mediator of inflammation affecting pathological new bone formation in AS. Targeting CaSR may be a novel potential therapeutic strategy to slow the progression of axial structural ankylosis.

## Introduction

Ankylosing spondylitis (AS) is a chronic inflammatory disease affecting the axial skeleton that is grouped under the term spondylarthritis (SpA) (Taurog *et al*, 2016; Sieper & Poddubnyy, 2017). In addition to inflammatory back pain and arthritic destruction, spinal ankylosis resulting from increasing pathological new bone formation is a typical feature of AS, causing disability and tremendous socioeconomic costs (Taurog *et al*, 2016). Although recent investigations and medications have focused on the aetiology and symptom‐controlling therapy, the pathological mechanism of pathological new bone formation is still not clarified. Targeted treatment of axial skeletal ankylosis is demanded because the prognosis so far is unsatisfying (Deodhar, 2018; Molnar *et al*, 2018).

The correlation between inflammation and pathological new bone formation remains enigmatic. Several studies have demonstrated that various types of osteogenic growth factors are involved in pathological new bone formation (Lories & Dougados, 2012; Ruiz‐Heiland *et al*, 2012; Gonzalez‐Chavez *et al*, 2016). Recently, we demonstrated that inflammation intensity‐dependent expression of osteoinductive factors and Wnt family proteins by proinflammatory cells plays a vital role in inflammation‐induced pathological new bone formation (Li *et al*, 2018). Thus, consistent with other studies, we confirmed that enhanced differentiation of osteogenic precursor cells induced by growth factors is an important molecular mechanism of inflammation‐induced bone formation in the pathological microenvironment. However, other than the indirect effect through induction of osteogenic growth factors, whether inflammatory cytokines have direct positive regulatory effects on osteogenic differentiation of precursor cells remains unknown.

Calcium‐sensing receptor (CaSR) is a diametric family of G protein‐coupled receptors, which have a critical role in modulating Ca^2+^ homeostasis via its role in the parathyroid glands and kidneys, which might affect bone mass. Normally, CaSR modulates systemic Ca^2+^ homeostasis by detecting increasing circulating concentrations of Ca^2+^, leading to intracellular signalling events that mediate reduced parathyroid hormone (PTH) secretion and a reduction in renal tubular Ca^2+^ reabsorption, therefore influencing skeletal homeostasis (Gowen *et al*, 2000; Hannan *et al*, 2016).

Moreover, CaSR is abundantly expressed in cells of osteogenic lineage and in skeleton tissues. Several studies have confirmed that CaSR plays a pivotal role in modulating differentiation and mineralization of osteogenic precursor cells (Chang *et al*, 2008; Hendy & Canaff, 2016; Hannan *et al*, 2018b). Activation of CaSR downstream pathways in osteoblasts promotes osteogenic differentiation *in vitro*, while ablating CaSR expression specifically in osteoblasts suppresses osteogenic activity and the mineralizing function in bony calluses and leads to decreased bone mass in animal models (Chang *et al*, 2008; Dvorak‐Ewell *et al*, 2011; Gonzalez‐Vazquez *et al*, 2014; Cheng *et al*, 2020).

Interestingly, previous studies showed that hyperstimulation of CaSR is a driving factor of the pathological progression in multiple inflammatory conditions, such as asthma and burn injury (Wu *et al*, 2015; Yarova *et al*, 2015; Cheng, 2016; Lee *et al*, 2017). Some studies reported that several inflammatory cytokines, including IL‐6 and IL‐1β, drive overexpression of CaSR and worsen the pathological condition in burn injury and sepsis (Hendy & Canaff, 2016; Klein *et al*, 2016). Given that activation of CaSR and downstream cellular signalling enhances osteogenic differentiation of osteoblasts, while aberrant expression of CaSR is commonly observed under inflammatory conditions, we speculated that CaSR might be involved in the pathological process of pathological new bone formation in the inflammatory microenvironment of AS.

In the current study, we explored the role of CaSR in AS and observed overexpression of CaSR in osteoblasts in spinal entheseal tissues collected from AS patients. Furthermore, aberrant expression of CaSR and activation of its downstream signalling pathways was also confirmed in several classic animal models of AS with different hypothetical types of pathogenesis. Inhibition of CaSR activation attenuated pathological new bone formation phenotype *in vivo* and osteogenic differentiation in cultured hBMSCs *in vitro*. Furthermore, various inflammatory cytokines were confirmed to enhance CaSR expression in osteoblasts. Hence, our findings revealed a prominent role for CaSR in the interplay between inflammation and the process of ankylosis progression, which might shed more light on the enigma of inflammation‐related pathological new bone formation in AS and propose a potential therapeutic target for slowing ankylosis progression.

## Results

### CaSR^+^ osteoblasts accumulate in spinal tissues from AS patients

**Figure 1:**
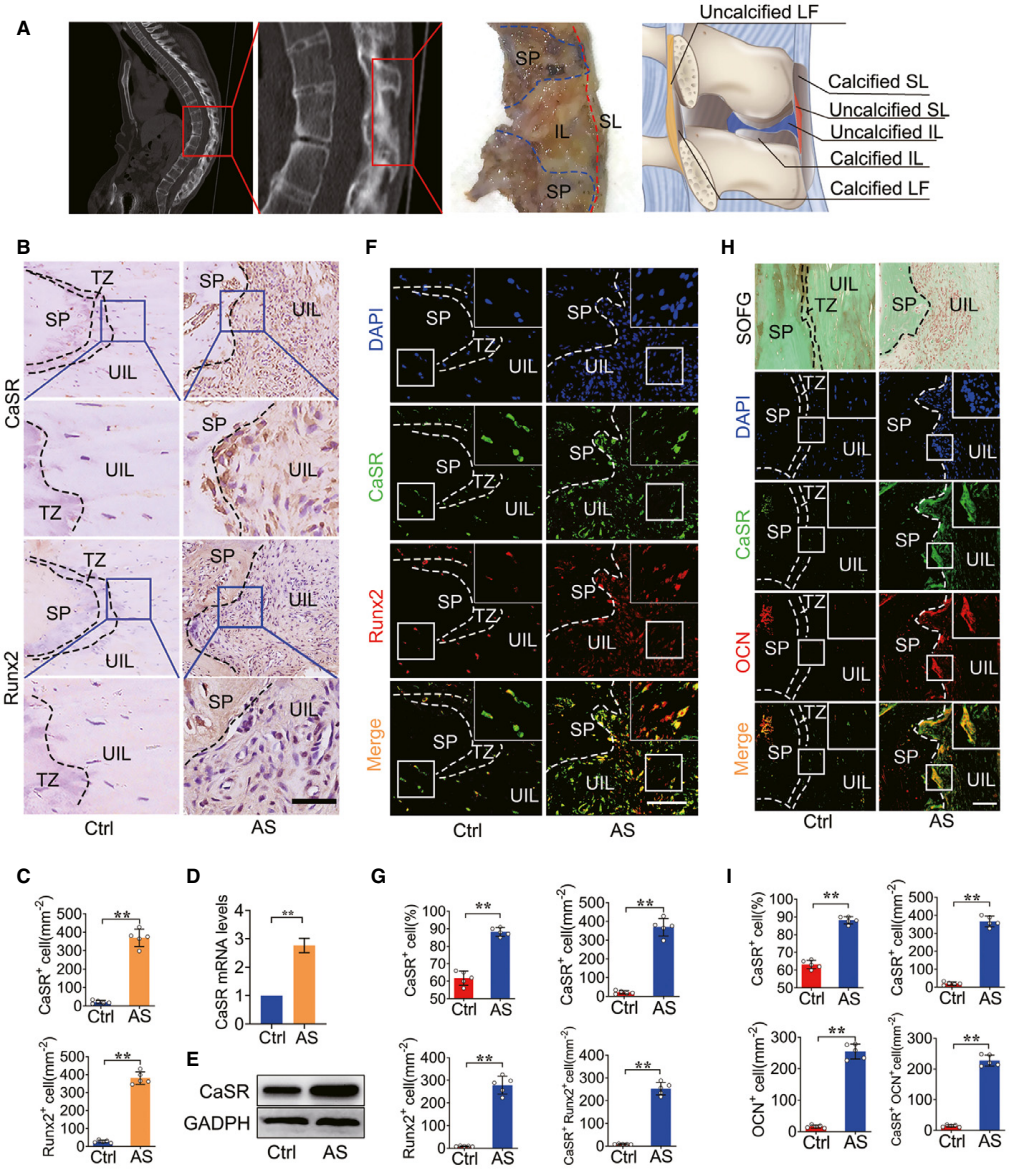
CaSR^+^ osteoblasts accumulated in the spinal entheseal tissues from AS patients An illustration of spinal ligament tissues collection.SOFG staining of spinal entheseal tissue and immunohistochemical analysis of CaSR^+^ and Runx2^+^ cells in human spinal tissue. The bottom panels show higher magnification of the boxed area in the top panels.Quantitative analysis of the number of CaSR^+^ and Runx2^+^ cells per area (mm^2^). *n* = 5, Student’s *t*‐test.RT–qPCR analysis of expression of CaSR in human spinal tissue. *n* = 3, Student’s *t*‐test.Western blot analysis of expression of CaSR in human spinal tissue.Immunofluorescence analysis of CaSR^+^ (Green) and Runx2^+^ (Red) cells in human spinal tissue.Quantitative analysis of CaSR^+^, Runx2^+^ and CaSR^+^ Runx2^+^ cells number per area (mm^2^) and CaSR^+^ cell percentage. *n* = 5, Student’s *t*‐test.Immunofluorescence analysis of CaSR^+^ (Green) and OCN^+^ (Red) cells in human spinal tissue.Quantitative analysis of CaSR^+^, OCN^+^ and CaSR^+^ OCN^+^ cells number per area (mm^2^). *n* = 5, Student’s *t*‐test. Data information: SP: spinous process; IL: interspinous ligament; SL: supraspinous ligament; UIL: uncalcified interspinous ligament; TZ: transitional zone. Data shown as mean ± SD. **P < 0.01 compared between groups. Scale bar: 100 μm.

To investigate whether CaSR plays a role in the process of pathological new bone formation in AS, we first assessed expression of CaSR in uncalcified spinal ligament tissues (supraspinous ligament and interspinous ligament) from AS patients who underwent correction surgeries (Fig 1A). Immunohistochemical staining showed that increased CaSR^+^ cells were accumulated at the entheseal site of the spinal ligament attaching the spinous process from patients with AS than in age‐ and sex‐matched controls. Cells that expressed Runx2, an osteogenic marker of naive osteoblasts, also accumulated at the same site as CaSR^+^ cells in the AS group (Fig 1B and C). RT–qPCR and Western blot analysis showed that expression of CaSR was increased in spinal ligament tissues of AS (Fig 1D and E). Immunofluorescence staining revealed that cells highly expressing CaSR were primarily Runx2^+^ naive osteoblasts (Fig 1F and G). SOFG (Safranine O‐Fast Green) staining showed that spinous process and calcified ligament were indistinguishable. Meanwhile, increased cells accumulated at the uncalcified zone in the AS group (Fig 1H). In addition, immunofluorescence staining of the same region showed that the majority of CaSR^+^ cells expressed OCN, a mature osteoblast marker (Fig 1H and I). These results suggested that CaSR^+^ osteoblasts accumulated at potential pathological new bone‐forming site of enthesis of the spinal ligament in AS patients.

### CaSR^+^ osteoblasts accumulate at pathological new bone‐forming sites in animal models of AS

**Figure 2:**
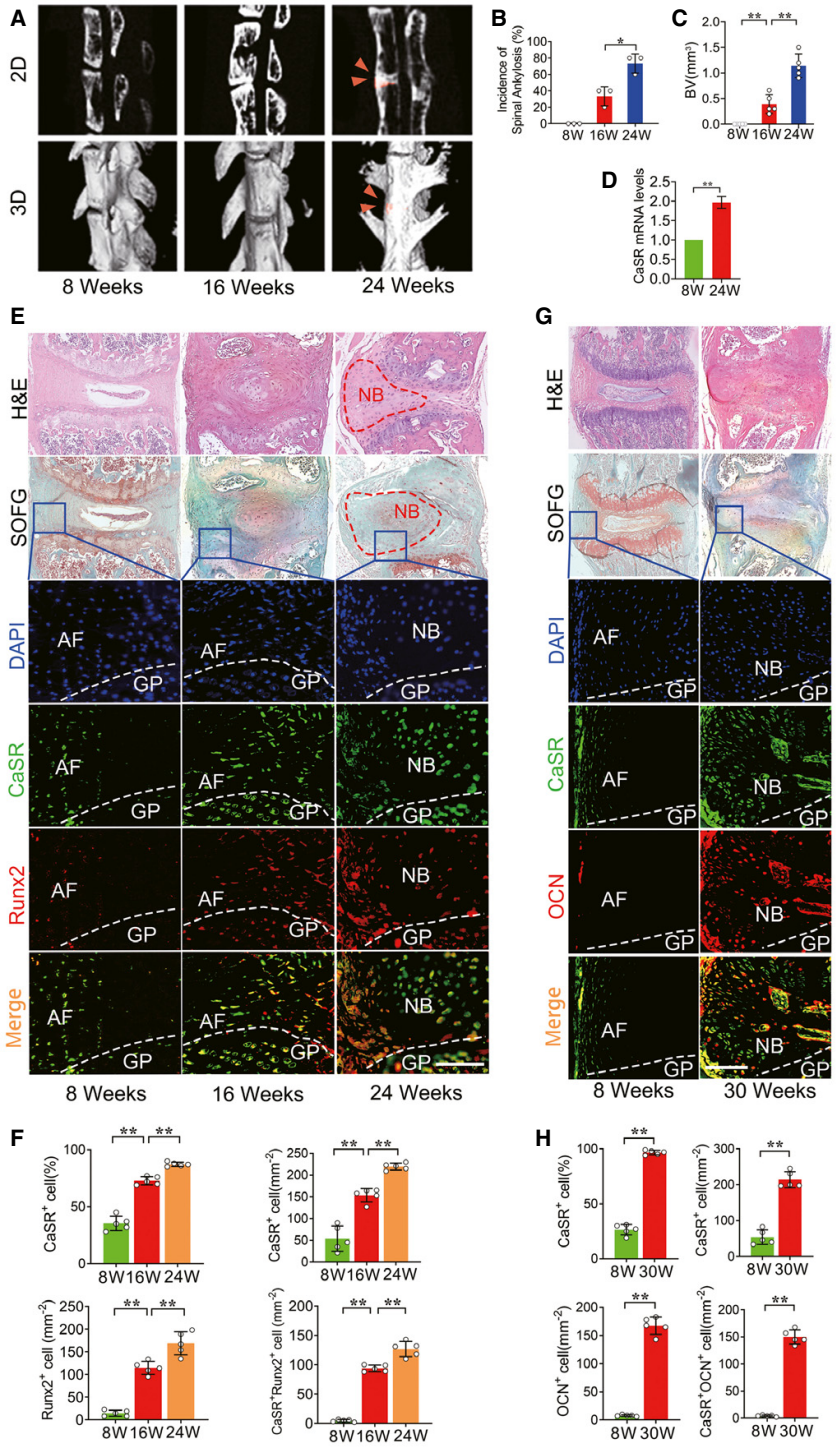
CaSR^+^ osteoblasts mediate new bone formation in PGIS model μCT images of the spine in PGIS model (2D and 3D reconstruction). Arrow head shows spinal ankylosis. *n* = 5 per group. μCT images of new bone (red area) in hind paws (2D and 3D reconstruction) in DBA/1 model compared to baseline.Incidence of spinal ankylosis in PGIS model. *n* = 5 per cage of total 3 cages per group, Fisher’s exact test.Quantitative analysis of structural parameters of spinal ankylosis by μCT analysis. *n* = 5 per group, one‐way ANOVA, Bonferroni *post hoc*.RT–qPCR analysis of expression of CaSR in spinal ligament tissue. *n* = 3, Student’s *t*‐test.H&E, SOFG and Immunofluorescence analyses of the spine specimen of PGIS model compare to baseline. *n* = 5 per group.Quantitative analysis of CaSR^+^, Runx2^+^ and CaSR^+^ Runx2^+^ cells number and CaSR^+^ cell percentage in the spine specimen of PGIS model. *n* = 5 per group, one‐way ANOVA, Bonferroni *post hoc*.H&E, SOFG and immunofluorescence analyses of the spine specimen of PGIS model at 30 weeks compared to baseline. GP: Growth plate. *n* = 5 per group.Quantitative analysis of CaSR^+^, OCN^+^ and CaSR^+^ OCN^+^ cells number in the spine specimen of PGIS model. *n* = 5 per group, one‐way ANOVA, Bonferroni *post hoc*. Data information: AF: Annulus fibrosus; GP: Growth plate; NB: New bone. Data shown as mean ± SD. **P < 0.01 compared between groups. Scale bar: 100 μm.

A proteoglycan‐induced spondylitis (PGIS) mouse model that exhibits both axial and peripheral inflammation was established to observe pathologic changes in spinal ankylosis (Hanyecz *et al*, 2004; Szabo *et al*, 2005; Adarichev & Glant, 2006; Berlo *et al*, 2006). The result of μCT analysis showed that spinal ankylosis gradually developed 24 weeks after PG induction (Fig 2A). The incidence of spinal ankylosis per cage increased from 0% to 33.3 ± 11.55% 8 weeks later and continued rising to 73.33 ± 11.55% at 24 weeks (Fig 2B). The bone volume (BV) of pathological new bone was increased 16 and 24 weeks after PG induction (Fig 2C). Meanwhile, RT–qPCR analysis showed that expression of CaSR was increased in spinal ligament tissues of the PGIS model (Fig 2D). H&E staining revealed pathological new bone formation at spinal entheseal sites attaching to the vertebral growth plate at 24 weeks. SOFG staining showed pathological process of new bone formation at 16 and 24 weeks through endochondral ossification (Fig 2E). Immunofluorescence staining revealed that cells expressing CaSR were primarily Runx2^+^ osteoblasts, which are analogous to human ligament tissues from AS patients. The number of CaSR^+^ Runx2^+^ osteoblasts was increased at spinal ligament tissues 16 and 24 weeks after PG induction compared to baseline (Fig 2E and F). Thirty weeks after PG immunization, most CaSR^+^ cells accumulated at spinal entheseal sites expressed OCN compared to baseline and 8 weeks (Fig 2G and H).

**Figure 3:**
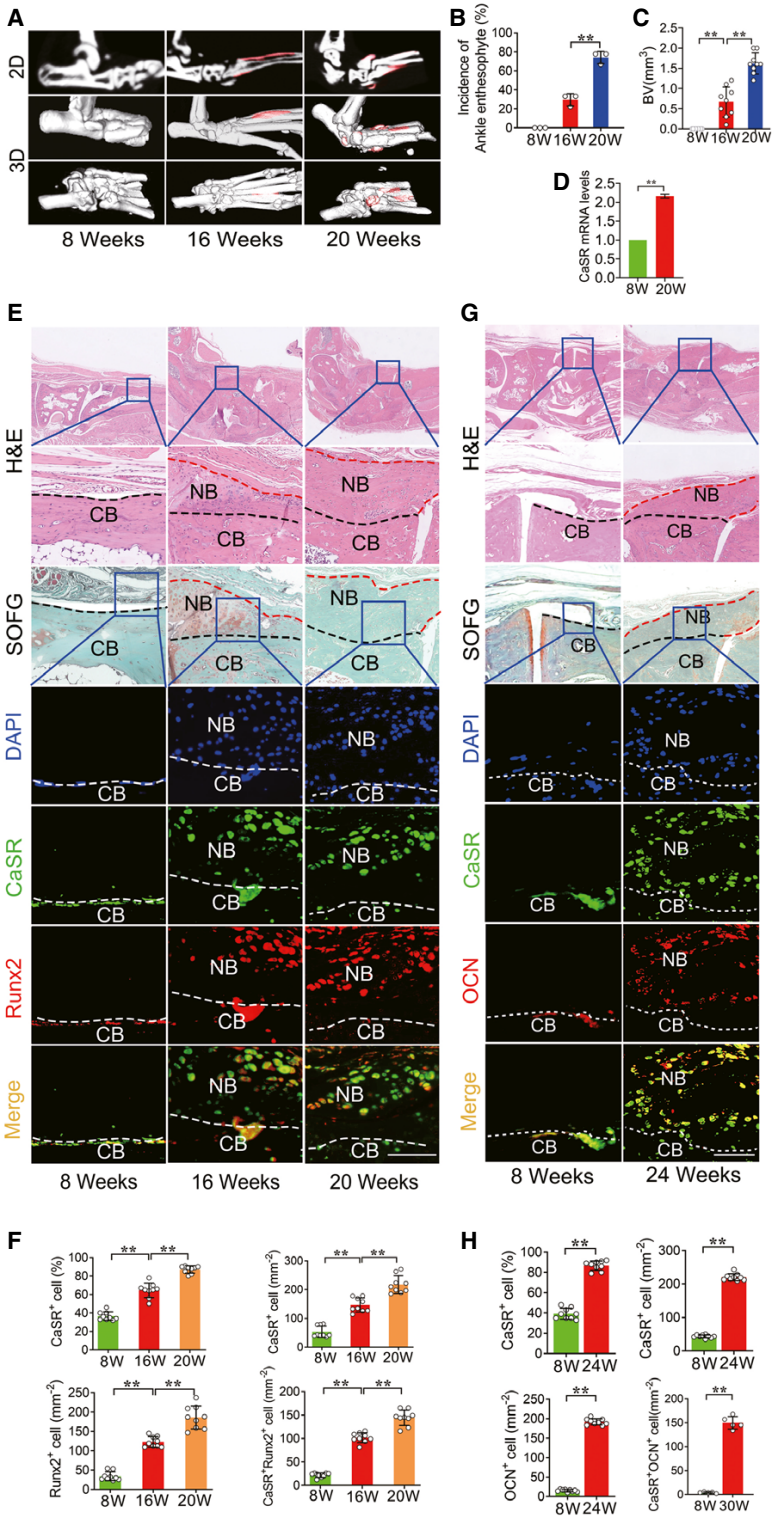
CaSR^+^ osteoblasts mediate new bone formation in DBA/1 model μCT images of pathological new bone formation in DBA/1 model. *n* = 9 per group.Incidence of ankle pathological new bone formation in DBA/1 model. *n* = 9 per cage of total 3 cages per group, Fisher’s exact test.Quantitative analysis of structural parameters of pathological new bone by μCT analysis. *n* = 9 per group, one‐way ANOVA, Bonferroni *post hoc*.RT–qPCR analysis of expression of CaSR in hind paw tissue. *n* = 3, Student’s *t*‐test.H&E, SOFG and immunofluorescence analyses of pathological new bone formation in hind paws. *n* = 9 per group.Quantitative analysis of CaSR^+^, Runx2^+^ and CaSR^+^ Runx2^+^ cells number and CaSR^+^ cell percentage in pathological new bone formation sites. *n* = 9 per group, one‐way ANOVA, Bonferroni *post hoc*.H&E, SOFG and immunofluorescence analyses of pathological new bone in hind paws. *n* = 9 per group.Quantitative analysis of CaSR^+^, OCN^+^ and CaSR^+^ OCN^+^ cells number in pathological new bone formation sites. *n* = 9 per group, one‐way ANOVA, Bonferroni *post hoc*. Data information: CB: Cortical bone; NB: New bone. Data shown as mean ± SD. **P < 0.01 compared between groups. Scale bar: 100 μm.

An ageing DBA/1 arthritis animal model spontaneously developed pathological new bone in the hind paws without spinal involvement compared to the PGIS animal model. This model shares multiple similarities with AS in humans, including enthesitis and entheseal new bone formation (Matthys *et al*, 2003). μCT analysis showed that pathological new bone formation (red area) in the hind paws was increased at 16 and 20 weeks of age (Fig 3A). The incidence of pathological new bone formation in the hind paws per cage increased from 0% at 8 weeks of age to 29.63 ± 6.41% and 74.08 ± 6.41% at 16 and 20 weeks of age, respectively (Fig 3B). BV of pathological new bone was gradually increased at 16 and 20 weeks of age (Fig 3C). RT–qPCR analysis showed that expression of CaSR was increased in hind paw tissues (Fig 3D). H&E and SOFG staining demonstrated pathological new bone formation at entheseal sites on dorsal surfaces at 16 and 20 weeks of age (Fig 3E). Immunofluorescence staining revealed that cells highly expressing CaSR were mostly Runx2^+^ osteoblasts analogous to the phenomenon observed in human ligament tissues from AS patients. The number of CaSR^+^ Runx2^+^ osteoblasts increased at entheseal sites on dorsal surfaces at 16 and 20 weeks of age (Fig 3E and F). At 24 weeks of age, most CaSR^+^ cells were accumulated at entheseal sites on dorsal surfaces and expressed OCN compared to baseline (Fig 3G and H).

**Figure EV1:**
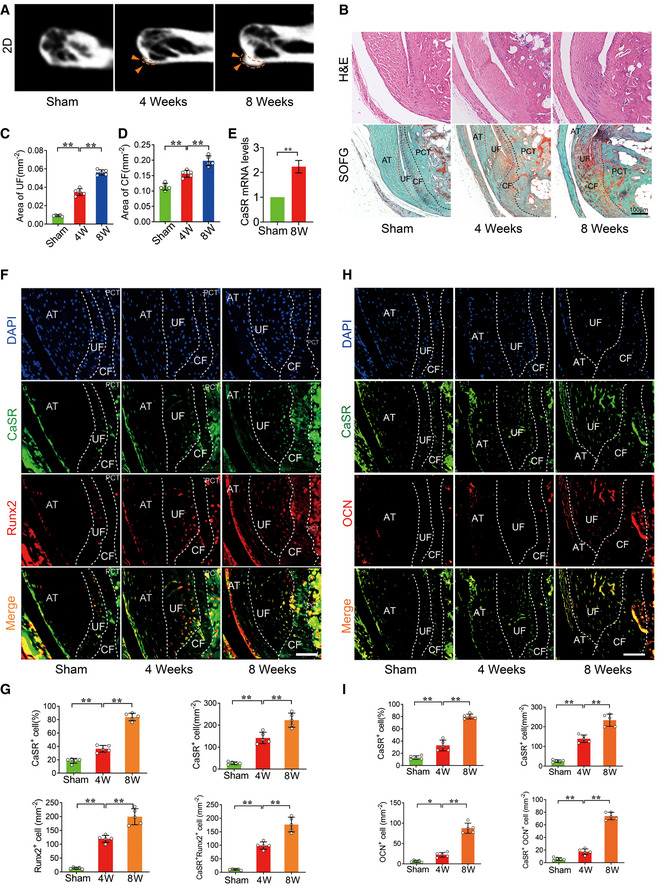
CaSR^+^ osteoblasts mediate pathological new bone formation of SMTS model μCT images of the PCT in SMTS model. Arrowhead shows bony projection. (2D).H&E and SOFG staining of Achilles tendon enthesis compartment in SMTS model. AT: Achilles tendon.Quantitative analysis of area of UF. *n* = 5, one‐way ANOVA, Bonferroni *post hoc.*
Quantitative analysis of area of CF. *n* = 5, one‐way ANOVA, Bonferroni *post hoc.*
RT–qPCR analysis of CaSR in SMTS model. *n* = 3, Student’s *t*‐test.Immunofluorescence analyses of Achilles tendon enthesis compartment in SMTS model.Quantitative analysis of CaSR^+^, Runx2^+^ and CaSR^+^ Runx2^+^ cells number and CaSR^+^ cell percentage in Achilles tendon enthesis compartment. *n* = 5, one‐way ANOVA, Bonferroni *post hoc.*
Immunofluorescence analyses of Achilles tendon enthesis compartment in SMTS model.Quantitative analysis of CaSR^+^, OCN^+^ and CaSR^+^ OCN^+^ cells number in Achilles tendon enthesis compartment. *n* = 5, one‐way ANOVA, Bonferroni *post hoc*. Data information: Data shown as mean ± SD. *P < 0.05, **P < 0.01 compared between groups. Scale bar: 100μm.

A semi‐Achilles tendon transection (SMTS) model was established to study pathological new bone formation due to the disruption of stress transmission at the posterior calcaneal tuberosity (PCT), as mechanical stress plays a prominent role in experimental AS (McClure, 1983; Jacques & McGonagle, 2014; Wang *et al*, 2018b). The results of μCT analysis showed that the entheseal bony projection gradually enlarged at 4 and 8 weeks compared to the sham‐operated group (Fig EV1A). H&E and SOFG staining revealed that areas of both uncalcified fibrocartilage (UF) (with rounded chondrocytes morphology) and calcified fibrocartilage (CF) (with calcified extracellular matrix and separated from UF by a tidemark) of the Achilles tendon were increased 4 and 8 weeks after surgery (Fig EV1B–D) (Raspanti *et al*, 1996; Hibino *et al*, 2007). RT–qPCR analysis showed that expression of CaSR was increased in entheseal tissues in SMTS model (Fig EV1E). Immunofluorescence staining demonstrated that cells highly expressing CaSR were mostly Runx2^+^ osteoblasts, and the number of CaSR^+^ Runx2^+^ cells increased in both UF and CF areas 4 and 8 weeks after surgery (Fig EV1F and G). At 4 and 8 weeks, a large amount CaSR^+^ cells had accumulated at the UF and CF of the Achilles tendon and expressed OCN, indicating CaSR^+^ mature osteoblasts were involved in the development of bony projections (Fig EV1Hand I).

These results suggested that CaSR^+^ osteoblasts might be involved in pathological new bone formation in AS animal models with different types of hypothetical pathogenesis (Vieira‐Sousa *et al*, 2015).

### Systemic inhibition of CaSR suppresses the ankylosing phenotype in animal models of AS

**Figure 4:**
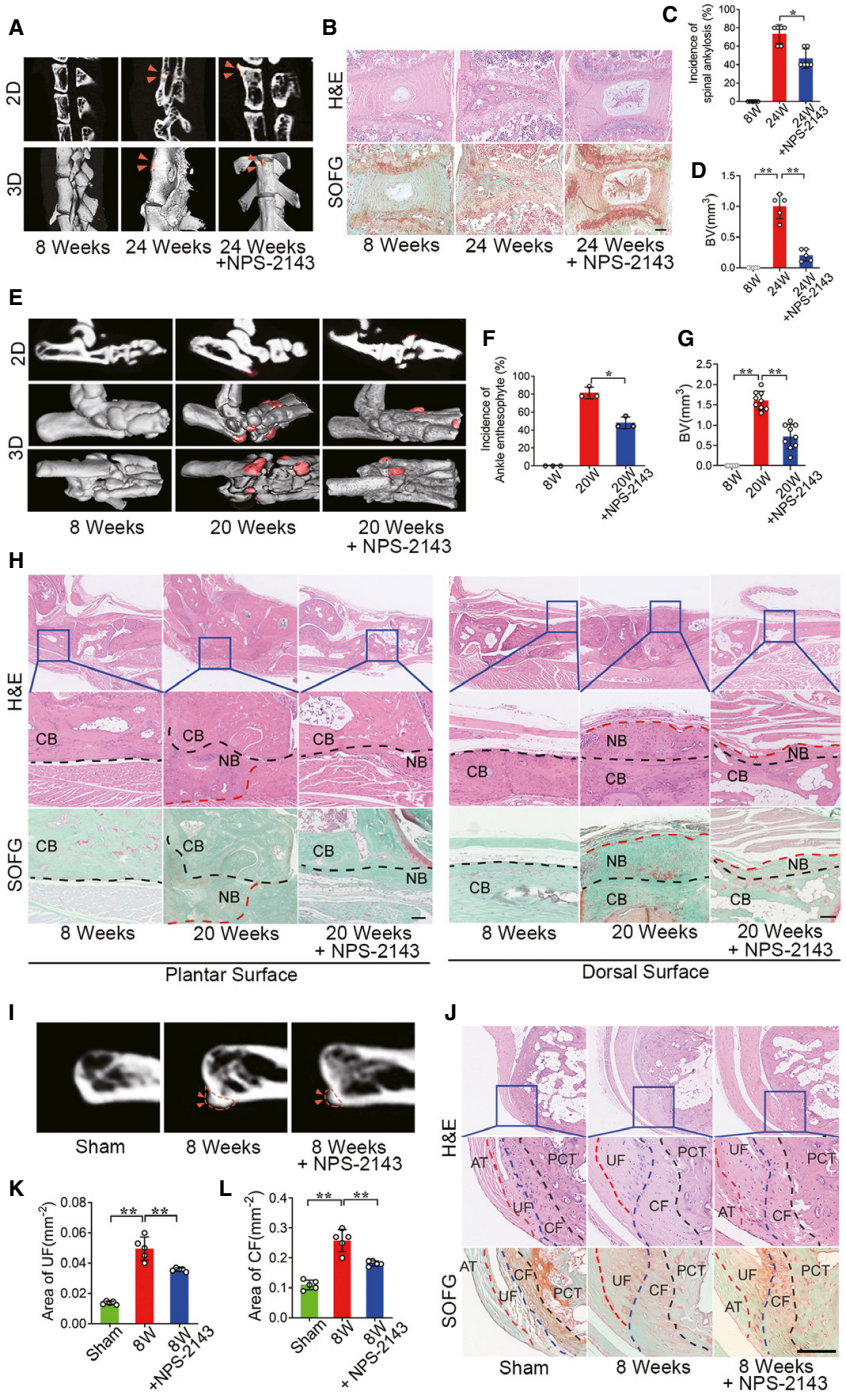
Systemic inhibition of CaSR suppresses the ankylosing phenotype of AS animal models μCT images of the spine in PGIS model (2D and 3D reconstruction). Arrow head shows spinal ankylosis.H&E and SOFG staining of spine in PGIS model.Incidence of spinal ankylosis in PGIS model. *n* = 5 per cage of total 6 cages per group, Fisher’s exact test.Quantitative analysis of structural parameters of new bone by μCT analysis. *n* = 5, one‐way ANOVA, Bonferroni *post hoc.*
μCT images of new bone formation (red area) in hind paws (2D and 3D reconstruction).Incidence of new bone formation in DBA/1 model. *n* = 9 per cage of total 3 cages per group, Fisher’s exact test.Quantitative analysis of structural parameters of new bone by μCT analysis. *n* = 9, one‐way ANOVA, Bonferroni *post hoc.*
H&E and SOFG staining of new bone in hind paws.μCT images of the PCT in SMTS model (2D). Arrow heads shows bony projection.H&E and SOFG staining of Achilles tendon enthesis compartment in SMTS model.Quantitative analysis of area of UF. *n* = 5, one‐way ANOVA, Bonferroni *post hoc.*
Quantitative analysis of area of CF. *n* = 5, one‐way ANOVA, Bonferroni *post hoc.* Data information: CB: Cortical bone; NB: New bone; UF: Uncalcified fibrocartilage; CF: Calcified fibrocartilage; PCT: posterior calcaneal tuberosity. Data shown as mean ± SD. **P < 0.01 compared between groups. Scale bar: 100 μm.

**Figure EV2:**
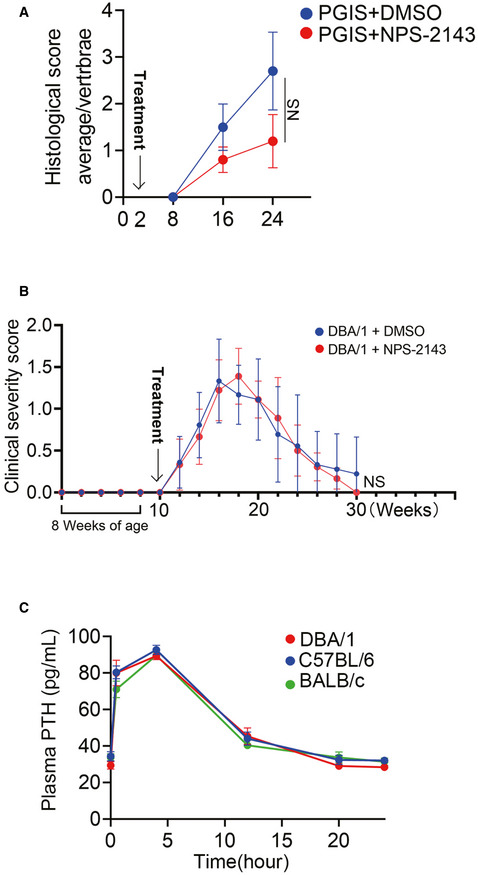
NPS‐2143 suppressed pathological new bone formation might be independent of PTH Histological score of PGIS mice. *n* = 5 per group, Mann–Whitney *U*‐test.Clinical severity scores of arthritis for DBA/1 mice. *n* = 9 per group, Repeated measures mixed models time*condition.Plasma PTH level in DBA/1, C57BL/6, and BALB/c mice after NPS‐2143 treatment. *n* = 3 per group. Data information: Data shown as mean ± SD. NS, not significant.

To validate the critical role of CaSR in pathological new bone formation, a selective CaSR antagonist, NPS‐2143, was administered systemically during the process of pathological new bone formation in these animal models. Following treatment with NPS‐2143 in PGIS, μCT analysis revealed that spinal ankylosis was ameliorated compared to the control group (DMSO administration) at 24 weeks (Fig 4A). H&E and SOFG staining demonstrated that spinal pathological new bone formation was attenuated after NPS‐2143 treatment (Fig 4B). Meanwhile, histological scores in the NPS‐2143 group were decreased at 16 and 24 weeks (Fig EV2A). The incidence of spinal ankylosis and pathological new bone formation was decreased compared to the control group (DMSO administration) at 24 weeks (Fig 4C and D). Similarly, following treatment with NPS‐2143 in the DBA/1 model, μCT analysis showed that pathological new bone formation (red area) in the hind paws was suppressed at 20 weeks of age (Fig 4E). Furthermore, the incidence of ankle pathological new bone formation was decreased compared to the control group (DMSO administration) at 20 weeks (Fig 4F and G). H&E and SOFG staining revealed that pathological new bone formation on both plantar and dorsal surfaces was decreased at 20 weeks of age in response to NPS‐2143 treatment (Fig 4H). However, the clinical severity score of the NPS‐2143 group did not decrease compared to the DMSO group (Fig EV2B). Similarly, in the SMTS model treated with NPS‐2143, μCT analysis demonstrated reduction of bony projections 8 weeks after surgery (Fig 4I). H&E and SOFG staining showed both UF and CF areas were decreased in SMTS mice in response to NPS‐2143 treatment (Fig 4J–L).

It has been proposed that inhibition of CaSR may stimulate parathyroid hormone (PTH) secretion, which might have an anabolic effect on bone formation (Gowen *et al*, 2000). To verify the effects of PTH, plasma PTH levels were measured, and results showed that plasma PTH levels peaked 4 h after dosing with NPS‐2143, gradually returning to baseline at 20 h in DBA/1, C57BL/6 and BALB/c mice (Fig EV2C).

These results strongly suggest that inhibition of CaSR suppresses pathological new bone formation in AS animal models due to different acknowledged types of hypothetical pathogenesis (Vieira‐Sousa *et al*, 2015).

### Suppression of the ankylosing phenotype by CaSR antagonists might involve the process of endochondral ossification

**Figure EV3:**
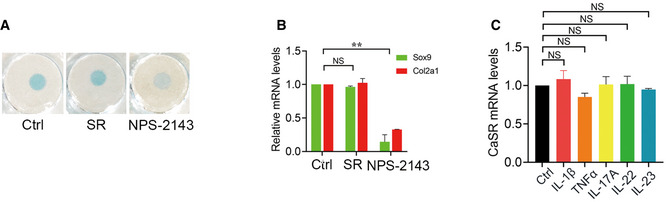
The role of CaSR in chondrocytes Alcian Blue staining of ATDC5 cells.RT–qPCR analysis of chondrogenic markers in ATDC5 cells. *n* = 3, one‐way ANOVA, Bonferroni *post hoc.*
RT–qPCR analysis of CaSR in ATDC5 cells. *n* = 3, one‐way ANOVA, Bonferroni *post hoc.* Data information: Data shown as mean ± SD. **P < 0.01 compared between groups, NS: not significant, *P* ≥ 0.05 compared between groups.

Since CaSR is also expressed in chondrocytes and plays a role in modulating chondrogenic differentiation and endochondral ossification, whether it affects the process of pathological new bone formation needed to be clarified. In our study, administration of a CaSR agonist (SR) failed to promote chondrogenic differentiation of murine chondrogenic ATDC5 cells. However, NPS‐2143 suppressed chondrogenic differentiation, as well as the transcription of chondrogenic marker genes, including Sox9 and Col2a1 (Fig EV3A and B). These results suggest that the inhibitory effect of NPS‐2143 in the ankylosing phenotype might involve inhibition of endochondral ossification.

### Activation of CaSR promotes osteogenic differentiation and pathological new bone formation through the PLCγ signalling pathway both *in vitro* and *in vivo*

**Figure 5:**
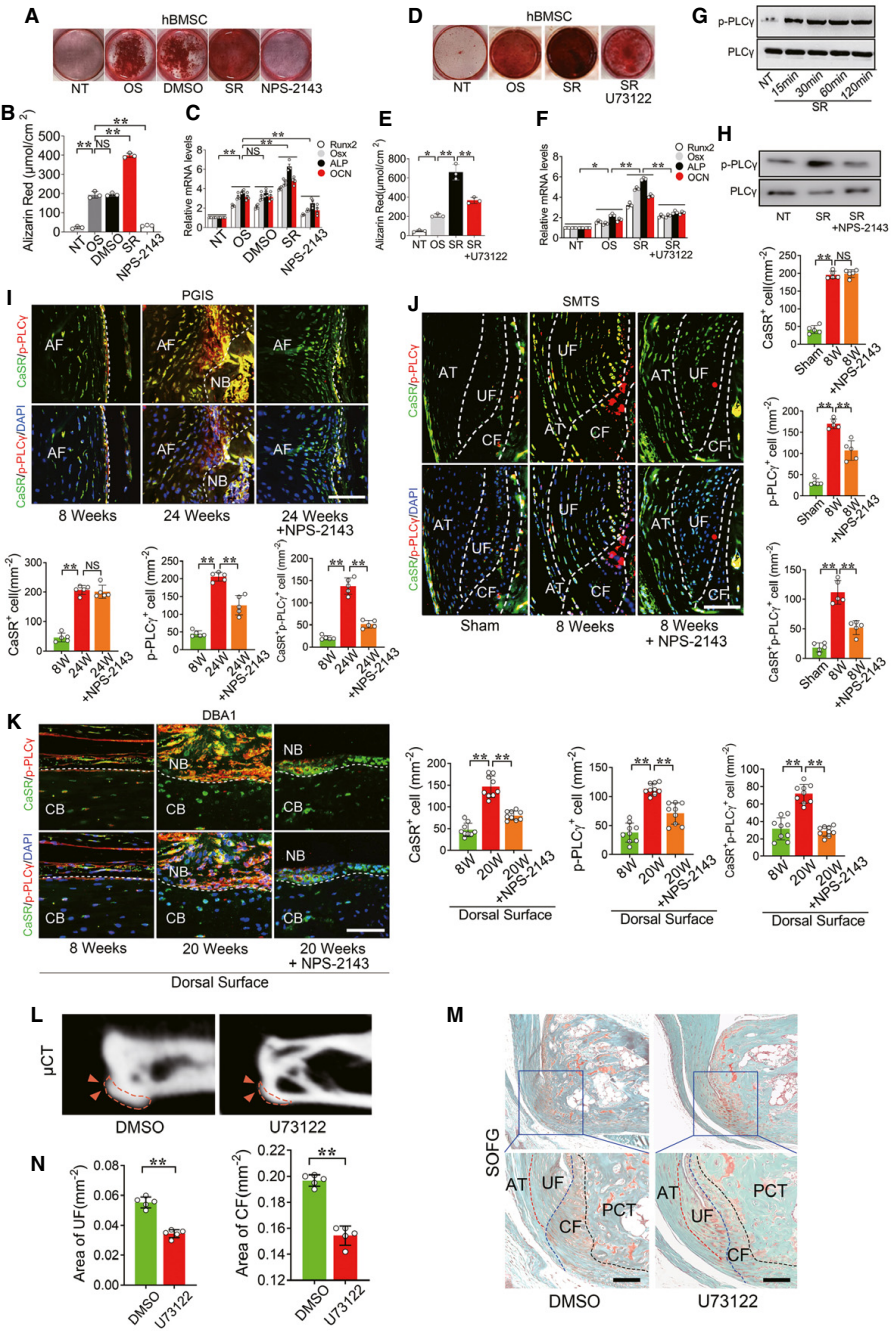
CaSR promotes osteogenic differentiation and new bone formation through PLCγ signalling pathway Alizarin Red staining of hBMSC cells for 11 days.Quantification of Alizarin Red staining at 570 nm (A570). *n* = 3, one‐way ANOVA, Bonferroni *post hoc.*
RT–qPCR analysis of osteogenesis markers in hBMSC cells. *n* = 3, one‐way ANOVA, Bonferroni *post hoc.*
Alizarin Red staining of hBMSC cells.Quantification of Alizarin Red staining at 570 nm (A570). *n* = 3, one‐way ANOVA, Bonferroni *post hoc.*
RT–qPCR analysis of osteogenesis markers in hBMSC cells. *n* = 3, one‐way ANOVA, Bonferroni *post hoc.*
Western blot analysis of phosphorylation of PLCγ protein levels in hBMSC cells.Western blot analysis of phosphorylation of PLCγ protein levels in hBMSC cells treated with SR and NPS‐2143.Immunofluorescence analysis of spine in PGIS model. Quantitative analysis of CaSR^+^, p‐PLCγ^+^ and CaSR^+^ p‐PLCγ^+^ cells number in spine of PGIS model. *n* = 5, one‐way ANOVA, Bonferroni *post hoc.*
Immunofluorescence analysis of Achilles tendon enthesis compartment in SMTS model. Quantitative analysis of CaSR^+^, p‐PLCγ^+^ and CaSR^+^ p‐PLCγ^+^ cells number in Achilles tendon enthesis compartment. *n* = 5, one‐way ANOVA, Bonferroni *post hoc.*
Immunofluorescence analysis of pathological new bone on dorsal surface of hind paws. Quantitative analysis of CaSR^+^, p‐PLCγ^+^ and CaSR^+^ p‐PLCγ^+^ cells number in pathological new bone formation sites. *n* = 9, one‐way ANOVA, Bonferroni *post hoc.*
μCT images of the PCT in SMTS model (2D). Arrow heads shows bony projection.SOFG staining of Achilles tendon enthesis compartment in SMTS model.Quantitative analysis of area of UF and CF. *n* = 5, Student’s *t*‐test. Data information: AF: Annulus fibrosus; CB: Cortical bone; NB: New bone; UF: Uncalcified fibrocartilage; CF: Calcified fibrocartilage; PCT: posterior calcaneal tuberosity. Data shown as mean ± SD. *n* = 5 for PGIS (I) and SMTS models (J); *n* = 9 for DBA/1 (K). *P < 0.05, **P < 0.01 compared between groups. Scale bar: 100 μm.

To determine the role of CaSR in osteogenic differentiation of hBMSCs, cells were pre‐treated with the CaSR‐specific agonist, strontium ranelate (SR), alone or in combination with the CaSR antagonist NPS‐2143 before osteogenic induction. Calcium deposition detected by Alizarin Red staining was increased in cells pre‐treated with SR and decreased in those pre‐treated with both SR and NPS‐2143 under osteogenic induction (Fig 5A and B). Similar effects on the expression of osteogenic marker genes, including Runx2, Osx, ALP and OCN, were observed 24 h after osteogenic induction (Fig 5C). These results suggest that CaSR might be critical for osteogenic differentiation in hBMSCs.

To further determine whether the PLCγ signalling pathway was involved in CaSR‐regulating osteogenic differentiation, hBMSCs were pre‐treated with a PLCγ specific inhibitor (U73122) before SR stimulation under osteogenic induction. Calcium deposition was partially but suppressed in hBMSCs (Fig 5D and E). Similar effects on expression of the osteogenic markers Runx2, Osx, ALP and OCN were observed at mRNA levels after osteogenic induction (Fig 5F). In addition, Western blot analysis showed that SR increased the phosphorylation of PLCγ (activated), and NPS‐2143 suppressed activation of PLCγ signalling by inhibiting CaSR (Fig 5G and H). These results suggest that CaSR promotes osteogenic differentiation through the PLCγ signalling pathway.

To confirm activation of the CaSR‐PLCγ signalling pathway *in vivo*, we observed phosphorylation of PLCγ in the established animal models. In the PGIS model, immunofluorescence staining demonstrated that positive staining of p‐PLCγ was notably increased in CaSR^+^ cells at the site of pathological new bone formation 24 weeks after induction, whereas NPS‐2143 treatment leads to reduction of positive of p‐PLCγ staining in CaSR^+^ cells (Fig 5I). In the SMTS model, immunofluorescence staining demonstrated that positive staining of p‐PLCγ was increased in CaSR^+^ cells at the Achilles tendon (AT), UF and CF 8 weeks after surgery, and NPS‐2143 treatment leads to reduced positive staining of p‐PLCγ in CaSR^+^ cells (Fig 5J). A similar effect on PLCγ signalling inhibition was observed in DBA/1 models on dorsal surfaces following NPS‐2143 treatment compared to activation of PLCγ signalling in the control group (Fig 5K). In the SMTS model administered U73122, μCT analysis revealed a reduction of bony projections 8 weeks after surgery (Fig 5 L). SOFG staining showed both UF and CF areas were decreased in SMTS mice in response to U73122 treatment (Fig 5M and N). These results suggest that CaSR promotes osteogenic differentiation and pathological new bone formation through PLCγ signalling both *in vitro* and *in vivo*.

### Multiple inflammatory cytokines induce CaSR upregulation in osteoblasts rather than chondrocytes

**Figure EV4:**
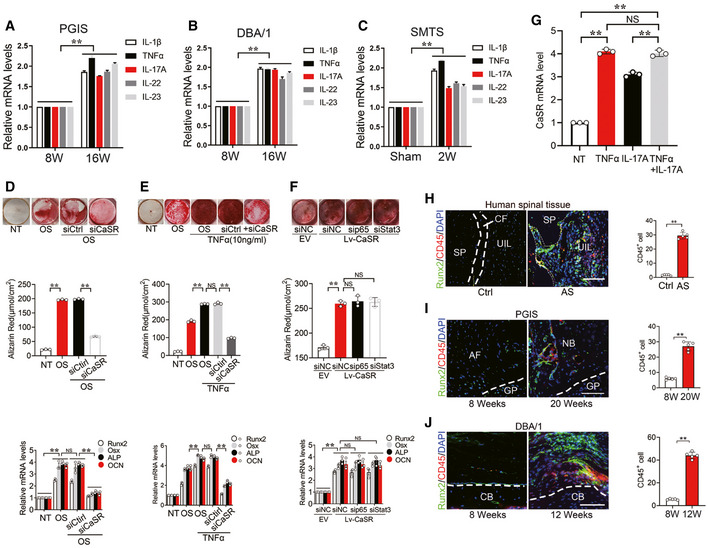
The interaction between CaSR and inflammation in pathological new bone formation A–CRT–qPCR analysis of CaSR mRNA level of entheseal tissues in PGIS, DBA/1 and SMTS models. *n* = 5, Student’s *t*‐test.DAlizarin Red staining of MC3T3‐E1 cells. Quantification of Alizarin Red staining and RT–qPCR analysis of osteogenesis markers of MC3T3‐E1 cells. *n* = 3, one‐way ANOVA, Bonferroni *post hoc.*
EAlizarin Red staining of MC3T3‐E1 cells. Quantification of Alizarin Red staining and RT–qPCR analysis of osteogenesis markers of MC3T3‐E1 cells. *n* = 3, one‐way ANOVA, Bonferroni *post hoc.*
FAlizarin Red staining of MC3T3‐E1 cells. Quantification of Alizarin Red staining and RT–qPCR analysis of osteogenesis markers of MC3T3‐E1 cells. *n* = 3, one‐way ANOVA, Bonferroni *post hoc.*
GRT–qPCR analysis of mRNA level of CaSR in MC3T3‐E1 cells. *n* = 3, one‐way ANOVA, Bonferroni *post hoc.*
HImmunofluorescence analyses of CD45^+^ and Runx2^+^ cells at spinal entheseal site in human tissues. Quantitative analysis of CD45^+^ cells. *n* = 5 per group, Student’s *t*‐test.IImmunofluorescence analyses of CD45^+^ and Runx2^+^ cells at spinal entheseal site in PGIS model. Quantitative analysis of CD45^+^ cells. *n* = 5 per group. Student’s *t*‐test.JImmunofluorescence analyses of CD45^+^ and Runx2^+^ cells at ankle entheseal site in DBA/1 model. Quantitative analysis of CD45^+^ cells. *n* = 5 per group. Student’s *t*‐test. Data information: Data shown as mean ± SD. *P < 0.01 or NS: *P* ≥ 0.05 compared between groups.

**Figure 6:**
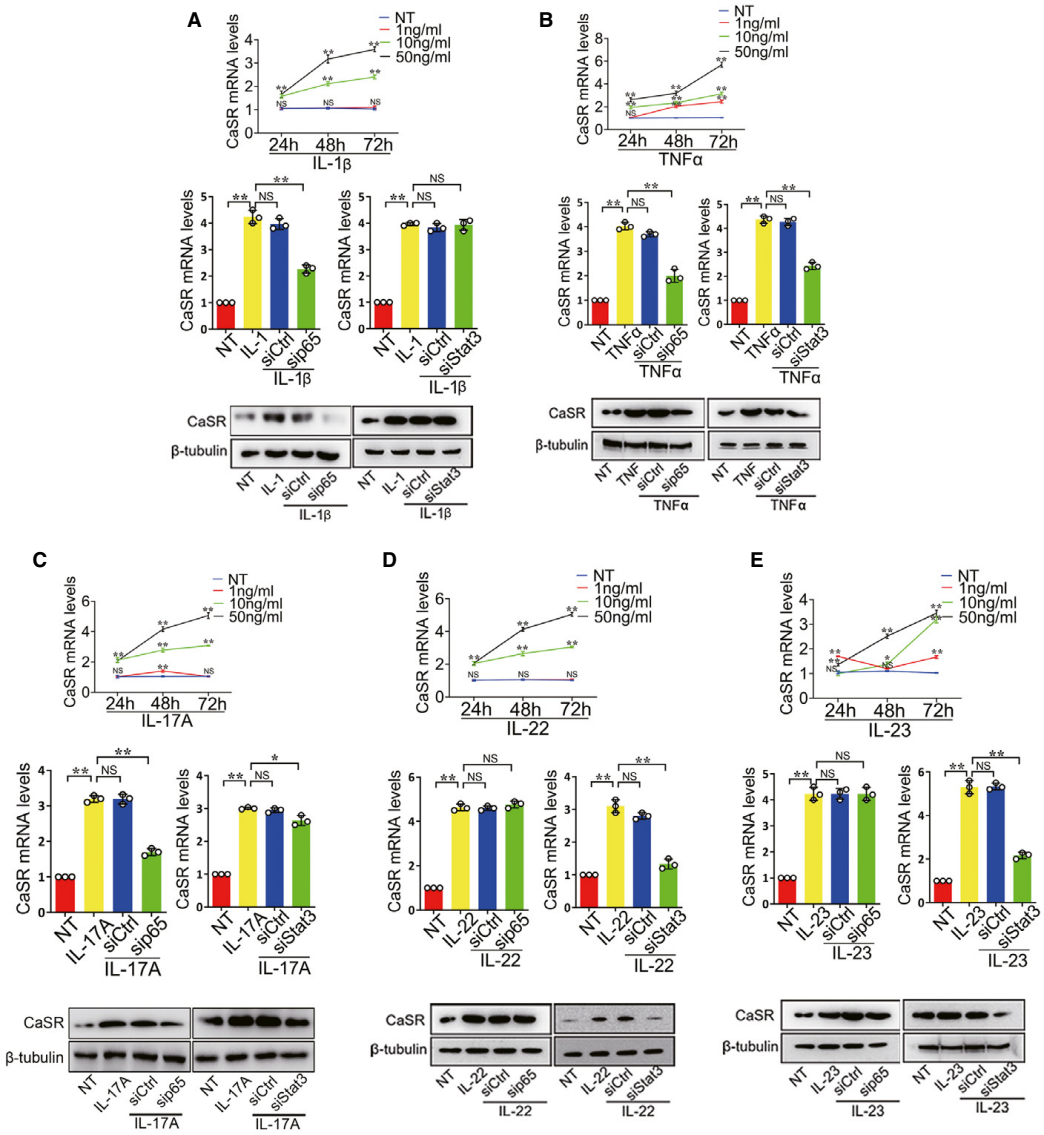
Multiple inflammatory cytokines induce CaSR upregulation in osteogenic precursor cells RT–qPCR and Western blot analyses of CaSR expressions in MC3T3‐E1 cells treated with IL‐1β. *n* = 3, one‐way ANOVA, Bonferroni *post hoc.*
RT–qPCR and Western blot analyses of CaSR expressions in MC3T3‐E1 cells treated with TNFα. *n* = 3, one‐way ANOVA, Bonferroni *post hoc.*
RT–qPCR and Western blot analyses of CaSR expressions in MC3T3‐E1 cells treated with IL‐17A. *n* = 3, one‐way ANOVA, Bonferroni *post hoc.*
RT–qPCR and Western blot analyses of CaSR expressions in MC3T3‐E1 cells treated with IL‐22. *n* = 3, one‐way ANOVA, Bonferroni *post hoc.*
RT–qPCR and Western blot analyses of CaSR expressions in MC3T3‐E1 cells treated with IL‐23. *n* = 3, one‐way ANOVA, Bonferroni *post hoc.* Data information: The data are presented as the means ± SD from one representative experiment of three independent experiments performed in triplicate. ^#^ *P* < 0.05, **P < 0.01 compared between groups.

Previous studies proved that multiple inflammatory cytokines, including IL‐1β, TNFα, IL‐17A, IL‐22 and IL‐23, are involved in pathological new bone formation in AS (van der Paardt *et al*, 2002; Sims *et al*, 2008; Sherlock *et al*, 2012; Tseng *et al*, 2016; Sieper & Poddubnyy, 2017; McGonagle *et al*, 2019; van Tok *et al*, 2019). Expression of IL‐1β, TNFα, IL‐17A, IL‐22 and IL‐23 was found increased in entheseal tissues from PGIS, DBA/1 and SMTS models (Fig EV4A–C). To determine whether upregulation of CaSR was induced by inflammatory cytokines, the MC3T3‐E1 pre‐osteoblast cell line was treated with inflammatory cytokines, including IL‐1β, TNFα, IL‐17A, IL‐22 and IL‐23. Results revealed that all theses inflammatory cytokines efficiently induced CaSR expression, confirmed by RT–qPCR and Western blot (Fig 6A–E). However, stimulation with these cytokines failed to induce upregulation of CaSR at the mRNA level in ATDC5 cells, indicating these inflammatory cytokines might not be able to induce upregulation of CaSR in chondrocytes (Fig EV2C).

To clarify the effect of CaSR on osteogenic differentiation in the inflammatory microenvironment, MC3T3‐E1 cells transfected with CaSR siRNA or control siRNA were cultured in osteogenic differentiation medium with or without low‐dose TNFα stimulation. The results showed that the osteogenic differentiation of the cells was suppressed by siCaSR treatment (Fig EV4D and E).

To further determine whether classic downstream signalling pathways of inflammatory cytokines regulate CaSR expression, the NF‐κB/p65 or JAK/Stat3 pathway was selectively inhibited by p65 or Stat3 target‐specific siRNA before inflammatory cytokine treatment. The results showed that by inhibiting the NF‐κB/p65 pathway, expression of CaSR in MC3T3‐E1 cells with IL‐1β, TNFα and IL‐17A stimulation was partially but significantly decreased (Fig 6A–C). Inhibition of the JAK/Stat3 pathway caused expression of CaSR to decrease at mRNA and protein levels partially but significantly in MC3T3‐E1 cells in response to TNFα, IL‐17A, IL‐22 and IL‐23 stimulation (Fig 6B, C and E). Overexpression of CaSR in MC3T3‐E1 cells promoted its osteogenic potential compared to negative control. Transfection with p65 or Stat3 siRNA did not affect the osteogenic effect of CaSR overexpression. These results indicate that the osteogenic effect of CaSR overexpression is independent of p65 or Stat3 pathways (Fig EV4F).

To validate whether TNFα and IL‐17A have a synergistic effect in upregulating CaSR expression during the process of pathological new bone formation, MC3T3‐E1 cells were treated with TNFα and IL‐17A alone or combination. RT–qPCR analysis of CaSR expression showed that upregulation of combined TNFα and IL‐17A was not significant compared to each treatment alone (Fig EV4G). These results suggest that multiple inflammatory cytokines induce CaSR upregulation in osteogenic precursor cells through NF‐κB/p65 and JAK/Stat3 signalling.

**Figure 7:**
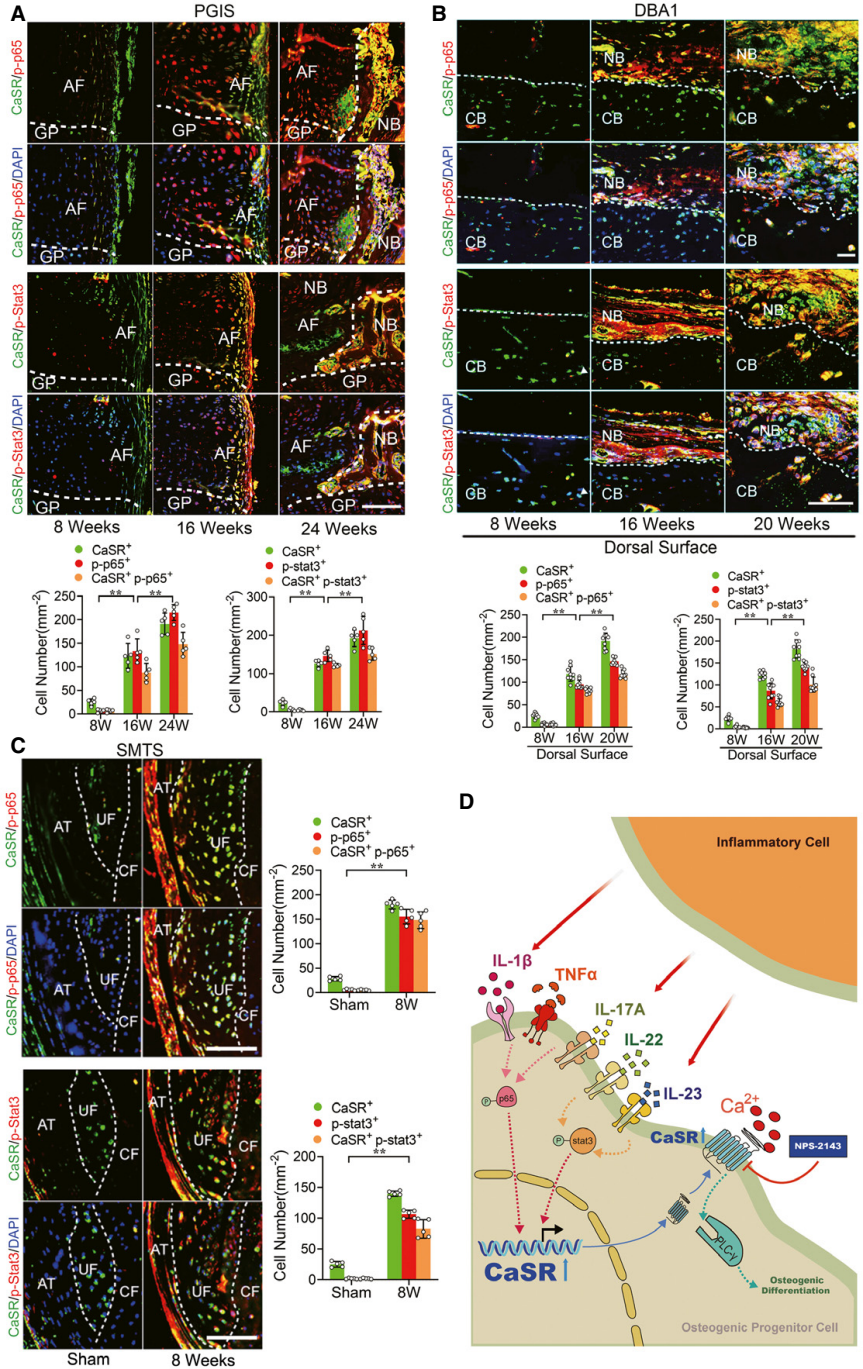
Inflammatory signalling pathways are activated in osteogenic precursor cells in AS animal models Immunofluorescence analysis of spine in PGIS mice. Quantitative analysis of CaSR^+^ p‐p65^+^ cells and CaSR^+^ p‐Stat3^+^ cells number in pathological new bone formation of PGIS model. *n* = 5, one‐way ANOVA, Bonferroni *post hoc.*
Immunofluorescence analysis of pathological new bone of hind paws in DBA/1 mice. Quantitative analysis of CaSR^+^ p‐p65^+^ cells and CaSR^+^ p‐Stat3^+^ cells number in pathological new bone formation sites. *n* = 9, one‐way ANOVA, Bonferroni *post hoc.*
Immunofluorescence analysis of Achilles tendon enthesis compartment in SMTS model. Quantitative analysis of CaSR^+^ p‐p65^+^ cells and CaSR^+^ p‐stat3^+^ cells number in PCT. *n* = 5, Student’s *t*‐test.Schematic presentation of multiple inflammatory cytokines from inflammatory cell upregulate hyperstimulation of CaSR through NF‐κB/p65 and JAK/Stat3 pathways. Hyperstimulation of CaSR induce osteogenic differentiation through PLCγ signalling pathway in osteogenic precursor cell to promote pathological new bone formation in AS. Data information: AF: Annulus fibrosus; GP: Growth plate; CB: Cortical bone; NB: New bone; UF: Uncalcified fibrocartilage; CF: Calcified fibrocartilage; PCT: posterior calcaneal tuberosity. Data shown as mean ± SD. **P < 0.01 compared between groups. Scale bar: 100 μm.

To confirm the activation of these inflammatory signalling pathways *in vivo*, we further examined the phosphorylation of p65 and Stat3 in the established animal models. In the PGIS model, immunofluorescence staining demonstrated positive staining of p‐p65 and p‐Stat3 was increased in CaSR^+^ cells in spinal ligament tissues 16 and 24 weeks after PG induction (Fig 7A). Similarly, in the DBA/1 model, immunofluorescence staining demonstrated positive staining of p‐p65 and p‐Stat3 was increased in CaSR^+^ cells at pathological new bone formation sites on dorsal surfaces at 16 and 20 weeks of age (Fig 7B). In the SMTS model, immunofluorescence staining demonstrated positive staining of p‐p65 and p‐Stat3 were increased in CaSR^+^ cells located at the AT, UF and CF 8 weeks after surgery (Fig 7C). In addition, CD45^+^ leukocytes accumulated around Runx2^+^ cells in tissue samples collected frome AS patients and animal models during the process of pathological new bone formation (Fig EV4H–J). The proximity of CD45^+^ cells and Runx2^+^ cells provides the opportunity for communication between these two cell types.

These results suggest that multiple inflammatory cytokines induce CaSR upregulation through NF‐κB/p65 and JAK/Stat3 signalling in osteogenic precursor cells to promote pathological new bone formation both *in vitro* and *in vivo* (Fig 7D).

## Discussion

Pathological new bone formation is a typical pathological feature in AS (Sieper & Poddubnyy, 2017). The correlation between inflammation and pathological new bone formation has so far not been clarified (Maksymowych *et al*, 2009; Pedersen *et al*, 2011; Lories & Dougados, 2012; Song *et al*, 2012; van der Heijde *et al*, 2012). Although it is acknowledged that inflammation induces secretion of osteogenic growth factors by immune cells and indirectly promotes pathological new bone formation at the enthesis, it is still unknown whether it has any positive regulatory effect on osteogenic differentiation of precursor cells in the same pathological new bone‐forming microenvironment (Ruiz‐Heiland *et al*, 2012; Li *et al*, 2018).

CaSR plays an essential role in bone homeostasis in both neonatal and adult stages (Goltzman & Hendy, 2015). Previous studies have shown that CaSR is also highly involved in many inflammatory diseases (Chang *et al*, 2008; Dvorak‐Ewell *et al*, 2011; Hendy & Canaff, 2016; Klein *et al*, 2016). However, whether it plays a role in pathological new bone formation in AS has not yet been reported. In the current study, we explored the role of CaSR in pathological new bone formation in AS and provided the first piece of evidence that CaSR expression was upregulated and CaSR + osteoblasts were accumulated in spinal ligament tissues collected from AS patients. These spinal ligament tissues were uncalcified tissues located right next to the calcified ligaments, which would potentially progress into calcified tissues and were considered bone‐forming sites (Lories & Haroon, 2017; Bruijnen *et al*, 2018; Wang *et al*, 2018b). Our finding from human specimens from AS patients indicated that aberrant upregulation of CaSR might play a critical role in osteogenic differentiation of precursor cells and subsequent pathological new bone formation at these locations (Fig 1).

The diversity of AS phenotypes, sharing peripheral, axial and extra‐articular manifestations (e.g. psoriasis, uveitis, and inflammatory bowel disease) with different degrees of severity, precludes the validity of using a single animal model for the study of human AS (Vieira‐Sousa *et al*, 2015). To confirm that CaSR was involved in pathological new bone formation *in vivo*, three AS animal models with different types of hypothetical pathogenesis were established. PGIS model is a systemic autoimmune murine model in which the abundance of autoantigens in this microenvironment initiates self‐sustaining immune reactions in susceptible strains that eventually lead to reactive new bone formation, ankylosis and spinal fusion (Bardos *et al*, 2005). So far, PGIS model is the unique reported model without genetically engineering that exhibits ankylosis phenotype in the spine to the best of our knowledge. Male DBA/1 mice spontaneously develop pathological new bone formation at entheses, which might be due to their susceptible genetic background, spontaneous inflammation, aggressive behaviour and sex‐related hormones (Holmdahl *et al*, 1992; Matthys *et al*, 2003). This animal model is widely used for the investigation of entheseal new bone formation (Vieira‐Sousa *et al*, 2015). In addition, mechanical loading has been recognized as an important factor that plays a critical role in enthesopathy and new bone formation, which is the typical pathological feather in AS (Jacques *et al*, 2014; Jacques & McGonagle, 2014; Ramiro *et al*, 2015; Schett *et al*, 2017; Wang *et al*, 2018b). It is hypothesized that mechanical stress and microdamage may be instrumental in inflammatory enthesitis as a primary driver (Jacques *et al*, 2014; Schett *et al*, 2017). To test whether aberrant upregulation of CaSR plays a critical role under this hypothesis, SMTS, a model with enthesopathy and pathological new bone formation due to unbalanced mechanical loading, was used (Wang *et al*, 2018b). Above all, our findings regarding CaSR from these animal models representing different hypotheses of pathogenesis of AS strongly suggested that aberrant upregulation of CaSR in osteoblasts is involved in pathological new bone formation in AS (Figs 1, 2, 3 and EV1).

To validate the critical role of CaSR in pathological new bone formation, a selective CaSR antagonist, NPS‐2143, which binds to the seven transmembrane domains and disrupts G protein‐mediated downstream signalling, was systemically administrated during the pathologic process of ankylosis in these animal models (Hannan *et al*, 2018b). Systemic administration of NPS‐2143 reduced the incidence of pathological new bone formation and the severity of the ankylosing phenotype in animal models with different types of hypothetical pathogenesis (Fig 4). In addition, ablation of CaSR suppressed osteogenic differentiation in the inflammatory environment (Fig EV4E). These findings indicate that CaSR plays a critical role in pathological new bone formation. Since it has been reported that NPS‐2143 modulates the secretion of PTH via interaction with CaSR in the parathyroid gland, this might change skeletal homeostasis (Gowen *et al*, 2000). Therefore, plasma PTH levels of experimental animals were determined. Results showed that PTH levels were upregulated immediately after NPS‐2134 application and sustained for approximately 6 h (twice the concentration compared to baseline), gradually reverting to baseline levels thereafter, in accordance with a previous report (Gowen *et al*, 2000). So far, no reported evidence has shown that systemic PTH levels are involved in pathological new bone formation in AS patients or animal models. Thus, reduced pathological new bone formation in response to NPS‐2134 may not be related to its effect on PTH secretion or its systemic regulation of skeleton homeostasis (Fig EV2C).

Previous studies and the current study suggest that both endochondral ossification and intramembrane ossification are important in pathological new bone formation in AS. CaSR plays a critical role in modulating chondrogenic differentiation, chondrocyte maturation and subsequent endochondral ossification (Goltzman & Hendy, 2015; Cheng *et al*, 2020). Knockdown of CaSR inhibited chondrogenesis of MSCs and hypertrophy and bone‐forming matrix secretion of chondrocytes (Cheng *et al*, 2020). However, hyperstimulation of CaSR did not enhance chondrogenic differentiation of MSCs. These findings indicate that CaSR is essential but not sufficient for endochondral ossification (Sarem *et al*, 2018). Consistent with previous studies, our results showed that CaSR antagonist NPS‐2143 suppressed chondrogenic differentiation of chondrogenic precursor cells. However, CaSR agonist SR failed to enhance chondrogenic differentiation in chondrogenic precursor cells (Fig EV3A and B). In addition, immune cytokines could upregulate expression of CaSR in osteoblasts but failed to increase the expression of CaSR in chondrocytes (Fig EV3C), indicating that inflammatory upregulation of CaSR mainly occurred in osteoblasts rather than chondrocytes during the process of pathological new bone formation. These results suggest that on one hand, inhibition of CaSR negatively impacted the process of endochondral ossification, meanwhile on the other hand directly suppressed intramembrane ossification.

Previous studies have noted that multiple downstream signalling pathways of CaSR participate in proliferation and osteogenic differentiation of osteoblasts, including PLCγ/PKC, MAPK (ERK1/2, JNK, p38) and cAMP/PKA (Yamaguchi *et al*, 2000; Godwin & Soltoff, 2002; Mizumachi *et al*, 2017). CaSR is reportedly linked to PLC via the heterotrimeric G protein isoforms Gq and mediates the downstream ERK1/2 pathway (Hannan *et al*, 2018a). PLC is a classic second massager of CaSR that is expressed in multiple organs, such as the parathyroid gland, vascular endothelium and vascular smooth muscle (Zhang *et al*, 2019; Ma *et al*, 2020). PLC protein mediates extracellular Ca^2+^ signalling and downstream biological functions, including hormone secretion, apoptosis and wound repair (Milara *et al*, 2010; Hannan *et al*, 2018a; Ma *et al*, 2020). Moreover, the PLCγ/PKC signalling pathway mediates osteogenic differentiation in osteoblasts (Moenning *et al*, 2009). Our findings demonstrated that the PLCγ pathway is involved in pathological new bone formation. Activation of PLCγ and subsequent pathological new bone formation was suppressed by the CaSR antagonist, NPS‐2143. These results suggest that the CaSR‐PLCγ signalling pathway is critical for pathological new bone formation *in vivo* (Fig 5). The above findings indicate that osteoblasts at entheseal sites may possess higher differentiation potential in the inflammatory microenvironment. Consistent with our results, some literature has stated that hBMSCs collected from AS patients differentiate towards bone nodules more efficiently than their non‐AS counterparts under osteogenic stimulation *in vitro* (Xie *et al*, 2016).

Another important finding in the current study is that multiple inflammatory cytokines, including TNFα, IL‐1β, IL‐17A, IL‐22 and IL‐23, promoted upregulation of CaSR in cultured osteoblasts in an independent manner. CD45^+^ immune cells accumulated around Runx2^+^ osteoblasts during the process of pathological new bone formation, providing an opportunity for communication between immune cells and osteoblasts (Fig EV4H–J). IL‐1β and IL‐17A induced CaSR primarily through the NF‐κB pathway, while IL‐22 and IL‐23 induced CaSR primarily through the JAK/Stat3 pathway. In addition, TNFα induced CaSR through both pathways (Figs 6 and 7). Recently, it has been reported that anti‐IL‐17 single application insufficiently reduced spinal pathological new bone formation in AS, while TNFi application during radiographic intervals in AS exerts minimal effect on spinal radiographic progression despite its clinically relevant inhibitory effect on spinal radiographic changes if applied before radiographic intervals (Sieper & Poddubnyy, 2016; Deodhar, 2018; Molnar *et al*, 2018; van Tok *et al*, 2019). Therefore, aberrant expression of CaSR independently induced by different inflammatory cytokines might partially explain the unsatisfying outcomes of either TNFi or anti‐IL‐17 administration. In the current study, although the CaSR antagonist did not suppress inflammation *in vivo* (Fig EV3B), it directly suppressed pathological new bone formation in animal models (Fig 4). These results indicate that suppression of CaSR and the involved downstream pathways might be more useful to the sufficient therapy for pathological new bone formation in AS.

There are certain limitations in the current study. First, although the focus of the current study was the aberrant expression of CaSR in osteoblasts, CaSR is also expressed in Runx2^−^ OCN^−^ cells in the pathological new bone‐forming area, and its role in other cell types in the pathological process remains unknown. An AS animal model established under the background of osteoblast‐specific ablation of CaSR should be the best method to specifically verify the effects of CaSR on pathological new bone formation. However, since the genetically modified animal models currently used are mostly established under the C57BL/6 background, some strains, such as BALB/c and DBA/1 mice, require backcrossing with CaSR*^flox/flox^* mice to establish genetically modified AS models for their own specific genetic background, which is considered critical for disease onset and progression. The biological effect of CaSR specifically in osteoblasts will be confirmed in our future work. Second, as CaSR is activated by extracellular Ca^2+^, the disadvantage of lacking a technique to detect dynamic changes in extracellular Ca^2+^ near pathological new bone formation sites needs further investigation. Third, CaSR is also reported to be involved in many inflammatory diseases (Hendy & Canaff, 2016; Klein *et al*, 2016). Whether augmentation or maintenance of inflammation by CaSR has a role in AS needs further investigations.

In summary, the novel findings of the current study suggest that inflammation‐induced aberrant upregulation of CaSR and activation of CaSR‐PLCγ‐signalling in osteogenic precursor cells acts as a mediator of inflammation affecting pathological new bone formation in AS. Targeting CaSR may be a novel potential therapeutic strategy to slow the progression of axial structural ankylosis in AS.

## Materials and Methods

### Patients

**Figure EV5:**
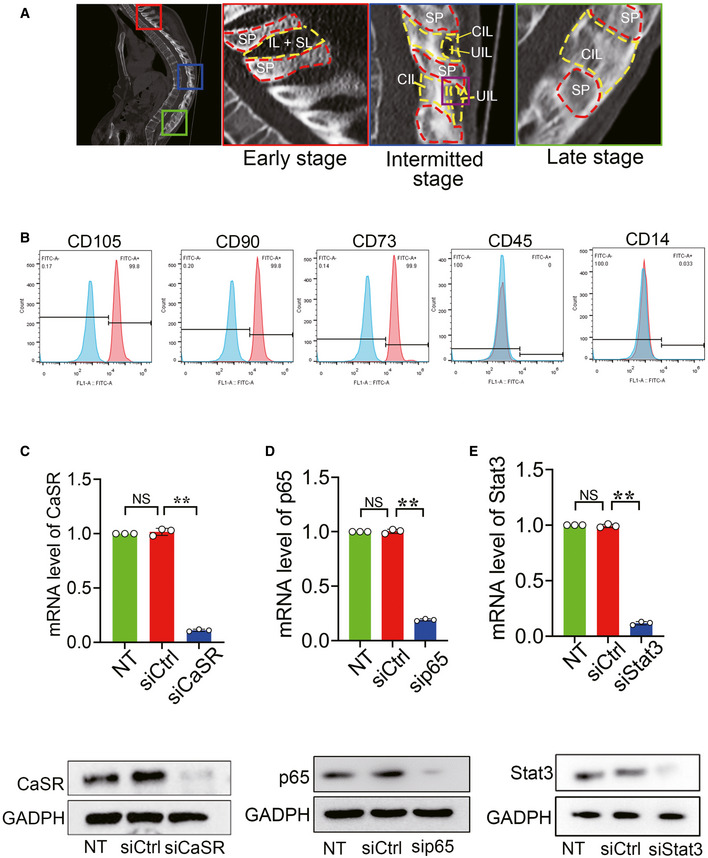
Functional verification of target‐specific siRNAs and identification of hBMSCs ARepresentative CT scanning of AS patient. SP: spinous process, IL: interspinous ligament; SL: supraspinous ligament; CIL: calcified interspinous ligament; UIL: uncalcified interspinous ligament (collected tissues).BhBMSCs exhibited positive staining for CD90, CD105 and CD73 but were negative for CD45 and CD14.C–ERT–qPCR and Western blot analyses of expression of Stat3, p65 and CaSR for functional verification of target‐specific siRNAs. *n* = 3, one‐way ANOVA, Bonferroni *post hoc.* Data information: Data shown as mean ± SD. **P < 0.01 compare between groups. NS: not significant.

Forty‐two patients (18 with AS and 24 with non‐AS) were consecutively enrolled at two hospitals between September 2013 and June 2019. AS patients who failed maintain a horizontal gaze in a natural, erect posture with the hips and knees comfortably extended fulfilled the criteria for deformity correction (Sciubba *et al*, 2008). AS patients with other diseases such as trauma, tumour, severe osteoarthritis in the lower limbs and a history of spine surgery were excluded from the study. Non‐AS patients fulfilled the criteria for correction of scoliosis or spinal decompression of thoracic or lumbar vertebrae (Lenke *et al*, 2001; Kreiner *et al*, 2013). Non‐AS patients were not known or suspected to have any systemic inflammatory condition including SpA (Appendix Table S1). In order to observe the molecular changes in the tissues that would potentially turn into calcified tissues, human spinal tissues obtained during corrective surgeries were the uncalcified tissues at the intermitted ossification stage (purple) (Fig EV5A). The Medical Ethics Committee of the First Affiliated Hospital of Sun Yat‐sen University approved the procedures performed in this study. Informed consent was obtained from all human subjects, and the experiments conformed to the principles set out in the WMA Declaration of Helsinki and the Department of Health and Human Services Belmont Report.

### Mice

Inbred, female BALB/c, male DBA/1 and C57BL/6J mice (8/9 weeks old) weighing between 18–22 g were purchased from the Charles River Laboratories. BALB/c, DBA/1 and C57BL/6J mice always housed on a 12h light/dark cycle with unrestricted access to food and water. Mice were randomly separated into each experiment group. For the PGIS model, BALB/c mice were mixed and caged in groups of 5 mice at 24 weeks of age. Cartilage PG was prepared as previous described (Mikecz *et al*, 1987; Tseng *et al*, 2016; Tseng *et al*, 2017). As a standard method, the first antigen injection (100 mg PG protein) was given in complete Freund’s adjuvant (Sigma, MO, USA), and the same doses of antigen were injected as second and third boosts in incomplete Freund’s adjuvant on weeks 3 and 6. For CaSR inhibition, the mice received treatment orally three times per week with NPS‐2134 (80 μmol/kg) (Sigma, MO, USA) or DMSO as negative control 2 weeks after third boost. Mice were euthanized at 8, 16 and 24 weeks after immunization. Spine specimens were dissected and fixed in 4% paraformaldehyde for histological analysis (*n* = 5 per group).

For the semi‐Achilles tendon transection (SMTS) model, C57BL/6J mice were semi‐transected the Achilles tendon to generate a destabilized enthesopathy animal model adjusted as in a previously described procedure (McClure, 1983; Wang *et al*, 2018b). Briefly, 3‐month‐old male C57BL/6J mice were anesthetized by ketamine and xylazine followed by semi‐transection of Achilles tendon in the middle of the tendon body to induce abnormal mechanical loading‐associated enthesopathy on the left calcaneus. Sham operations were done on independent mice. For CaSR inhibition, the mice received treatment orally three times per week with NPS‐2134 (80 μmol/kg) (Sigma, MO, USA) or DMSO as negative control 3 days after surgery. For PLCγ inhibition, the mice were intraperitoneally injected three times per week with 1 mg/kg U73122 (Sigma, MO, USA) or DMSO as negative control 3 days after surgery. Mice were euthanized at 4 and 8 weeks after immunization. Hind paw specimens were dissected and fixed with 4% paraformaldehyde for histological analysis (*n* = 5 per group).

For spontaneous‐arthritis DBA model, male DBA/1 mice were mixed and caged together in groups of 9 mice to induce arthritis at the age of 8 weeks (Lories *et al*, 2004; Lories *et al*, 2005; Li *et al*, 2018). Both hind paws were evaluated, resulting in a maximum score of 8. For CaSR inhibition, the mice received treatment orally three times per week with NPS‐2134 (80 μmol/kg) (Sigma, MO, USA) or DMSO as a negative control 2 weeks after caging. Mice were euthanized at the age of 8, 16 and 20 weeks. Hind paw specimens were dissected and fixed with 4% paraformaldehyde for histological analysis (*n* = 9 per group). Mice that have severe disease and resulted in> 15% body weight loss will be killed and excluded. In the current study, no mice reached the humane end points.

The Medical Ethics Committee of the First Affiliated Hospital of Sun Yat‐sen University approved the procedures performed in this study.

### Evaluation of severity of arthritis

DBA/1 mice were scored twice a week for clinical signs of arthritis as follows: Clinical score was graded per paw as follows: 0, normal; 1, swelling of one digit; 2, swelling of two or more digits; or 3, swelling of the entire paw. Scores were evaluated in a blinded manner as described previously (Sherlock *et al*, 2012).

Severity of vertebral joint disease was scored as described previously (Bardos *et al*, 2005); score 1, enthesitis, inflammatory cell accumulation around the IVD and/or infiltration of the annulus fibrosus; score 2, < 50% absorption/erosion of the IVD; score 3, essentially complete resorption (> 50%) of the IVD; score 4, cartilaginous/bony ankylosis.

### Biochemical assays

The concentrations of PTH in plasma were measured using Mouse Parathyroid Hormone EIA Kit (RayBio).

### Flow cytometric analysis

For identification of hBMSCs, cells were incubated with the monoclonal antibodies: FITC‐conjugated CD90 (1:50), FITC‐conjugated CD105 (1:50), FITC‐conjugated CD14 (1:50), FITC‐conjugated CD45 (1:50) and FITC‐conjugated CD73 (1:50). The cells were analysed by flow cytometry using CytoFLEX (Beckman Coulter). Data were acquired as the fraction of labelled cells within a single‐cell gate set of 10,000 events (Fig EV5B).

### Cell treatments

To induce osteogenesis, different progenitor cells were plated in a 6‐well plate at a density of 2 × 10^5^ cells/well and cultured for 24 h. The cells were then switched to osteogenic medium consisting of α‐minimum essential medium supplemented with 10% foetal bovine serum (FBS), 50 μg/ml l‐ascorbic acid, 0.1 μM dexamethasone and 10 mM b‐glycerophosphate to induce osteogenesis. The medium was changed every 3–4 days. To assess inflammatory stimulation, 10 ng/ml TNFα was added in osteogenic medium.

To assess chondrogenic differentiation, 100,000 ATDC5 cells (murine chondrogenic cell line) (Riken Cell Bank (Tsukuba, Japan)) were resuspended in 10 μl of control medium and seeded as micromasses in the middle of a 24‐well plate. Cells could attach for 1 h at 37°C, after which 0.5 ml of control, DMEM containing 10 ng/ml recombinant mice TGFβ1 (PeproTech), 50 μM l‐ascorbic acid 2‐sulphate (Sigma‐Aldrich) were added to the wells. Medium was refreshed every other day, and after 9 days, micromasses were either stained with alcian blue or used for RNA isolation.

To evaluate the effect of NPS‐2143 during osteogenesis or chondrogenesis, NPS‐2143 (0.1 μM) was added 1 h before strontium ranelate (SR, 1 mM) stimulation. To evaluate the effect of PLCγ pathway, U73122 (2 μM) was added 1 h before strontium ranelate (SR, 1 mM) stimulation. To investigate the effect of IL‐1β, TNFα, IL‐6, IL‐17A, IL‐22 and IL‐23 on CaSR expression, 1, 10 and 50 ng/ml of these inflammatory cytokines were added on MC3T3‐E1 cells. To investigate the effect of these inflammatory cytokines on CaSR expression in chondrocytes, 10 ng/ml of these inflammatory cytokines was added on ATDC5 cells.

### RNA extraction and quantitative real‐time PCR

Tissue samples were flash‐frozen in liquid nitrogen and stored at −80°C. Samples were homogenized separately in TRIzol (Life Technologies, Mulgrave, Victoria, Australia). For gene expression analysis, Total RNA was extracted from cells according to the manufacturer’s protocol, and 2 µg of total DNA‐free RNA was used to synthesize cDNA using the ReverTra Ace qPCR RT Kit (Toyobo, Osaka, Japan). The reactions were set up in 96‐well plates using 1 µl cDNA with Thunderbird SYBR qPCR Mix (Toyobo, Osaka, Japan), to which gene‐specific forward and reverse PCR primers were added. qRT–PCR was performed under the following conditions: 95°C for 10 min, followed by 40 cycles of 95°C for 10 s and 55°C for 34 s. Analysis was performed to detect CaSR, p65, Stat3, Runx2, Osx, ALP, OCN, Sox9 and Col2a1 expression, and β‐actin was used as an internal control. Primer sequences were as Appendix Table S2.

### Western blot

Spinal tissue protein was extracted with T‐PER tissue protein extraction reagent (Thermo Scientific), following the instructions of the manufacture. After protein extraction, protein concentration was determined with a BCA assay. A 10% SDS–PAGE gel was loaded with 20 µg of total protein, and the separated proteins were transferred by electro blotting to PVDF membranes. The membranes were blocked with 5% non‐fat dry milk in TBST (50 mM Tris, pH 7.6, 150 mM NaCl, 0.1% Tween 20) and incubated with the primary antibody overnight at 4°C in 5% non‐fat dry milk in TBST. Immunolabelling was detected using ECL reagent (Invitrogen, CA). The antibodies used for Western blot were from following source: anti‐CaSR antibody (Abcam, UK; 1:1,000), anti‐PLCγ antibody (Cell Signaling Technology, MA; 1:1,000), anti‐phospho‐PLCγ antibody (Cell Signaling Technology, MA; 1:1,000), anti‐β‐actin antibody (Sigma‐Aldrich, MO; 1:10,000) and anti‐β‐tubulin antibody (Sigma‐Aldrich, MO; 1:10,000).

### RNA interference

The small interfering RNA(siRNA) duplexes were pre‐designed with the online software (Stealth RNAi Pre‐Designed siRNAs) provided by Ambion (Thermo Scientific^TM^) (http://www.thermofisher.com/cn/zh/home/life-science/rnai/synthetic-rnai-analysis/stealth-select-rnai.html) and constructed by GenePharma (GenePharma Co., Suzhou, China). siRNA duplexes were presented in Appendix Table S3. The siRNAs would be verified to be efficient before all experiments (Fig EV5C–E). Cells were plated at a concentration of 1x10^5^ cells/well in 6‐well plates and transduced with the small interfering RNA (siRNA) using lipofectamine RNAiMAX transfection reagent (Invitrogen, USA) according to the manufacturer’s instructions. Different amounts of 20 μM siRNA duplexes were mixed with 5 μl/well of transfection reagent, and Opti‐MEM reduces serum medium (Invitrogen, USA) to total volume of 500 μl and incubated for 20 min. The mixture was applied to cell 16 h at 37°C in 5% CO_2_.

### Establishment of a stable CaSR‐overexpression cell line

MC3T3‐E1 cells were cultured in αMEM, and the plasmid carrying the CaSR gene and three helper plasmids were transferred into MC3T3‐E1 cells by lentivirus infection, and the CaSR gene was inserted into the genome of the cells. GFP‐positive cells were selected by flow cytometry. Monoclonal clones were selected to inoculate a 96‐well plate. Positive clones were transferred to a 12‐well plate to expand culture and appropriate CaSR overexpressing stable cell lines were selected according to the expression level. At the same time, cell lines with empty plasmids were constructed.

### Mineralization analysis

For detecting the mineralization, we used OS to induced MC3T3‐E1 or hBMSCs for 11 days. Cells were washed three times with PBS and fixed with 70% ethanol for 10 min. After three washes with distilled water, the cells were stained with a 40 mM alizarin red S (Sigma‐Aldrich) solution (pH 4.1) for 10 min to visualize matrix calcium deposition. The remaining dye was washed three times with distilled water, and the stained cells were photographed. For quantification, the calcium deposits were distained with 10% cetylpyridinium chloride in 10 mM sodium phosphate (pH 7.0), then the extracted stain was transferred to a 96‐well plate, and the absorbance of the samples was measured at 570 nm using a microplate reader (Tecan, Salzburg, Austria) (Li *et al*, 2018; Li *et al*, 2019).

### Immunofluorescence

Tissue sections were fixed in 4% PFA for 30 min and permeabilized with 0.3% Triton X‐100 for 30 min. Blocking was performed with 5% normal goat serum for 1 h. The tissue sections and the cells were incubated overnight at 4°C in the primary antibodies against following antigens: anti‐CaSR antibody (Abcam, UK; 1:200), anti‐Runx2 antibody (Abcam, UK; 1:200), anti‐p‐PLCγ antibody (Abcam, UK; 1:200), anti‐p‐p65 antibody (Abcam, UK; 1:200), anti‐p‐Stat3 antibody (Abcam, UK; 1:200), anti‐CD45 antibody (Abcam, UK; 1:100) and anti‐Col2a1 antibody (Abcam, UK; 1:100). After washing three times in PBS, the primary antibodies were probed with the secondary antibodies Alexa Fluor 594 goat anti‐rabbit (1:500, Invitrogen, Camarillo, CA) and Alexa Fluor 488 goat anti‐mouse (1:500, Invitrogen) for 1 h at room temperature. Finally, the coverslips were washed in PBS three times and mounted using Prolong Gold Antifade Reagent containing 4′‐6‐diamidino‐2‐phenylindole (DAPI) (Molecular Probes, Invitrogen). The targeted marker‐positive cells in each visual field were counted under a fluorescence microscope (Carl Zeiss Axio Observer Z1, Zeiss, Oberkochen, Germany). Data were acquired and analysed in a double‐blinded manner. The investigators who performed the analysis comprised a separate group from investigators who performed immunofluorescence staining, and the two separate groups worked without knowing the different treatments designed by the principle investigator.

### μCT and histologic analyses

All specimens were obtained from mice post‐mortem and fixed with 4% paraformaldehyde. For μCT scanning, specimens were fitted in a cylindrical sample holder and scanned using a Scanco lCT40 scanner set to 55 kVp and 70 lA. For visualization, the segmented data were imported and reconstructed as three‐dimensional images using MicroCT Ray V3.0 software (Scanco Medical).

For histologic analysis, specimens were decalcified in 0.5M EDTA (Sigma‐Aldrich) at 4°C. Paraffin‐embedded sections were stained with haematoxylin and eosin (H&E) and Safranin O‐Fast Green (SOFG) to evaluate general structures and bone formation. Immunohistochemical analysis of the specimens was conducted using specific antibodies. Tissue sections were quantitated according to the number of positive cells in per area as previously described (Li *et al*, 2018; Wang *et al*, 2018a, 2018b).

### Statistics

Data obtained from experiments in duplicate or triplicate and repeated at least three times were represented as mean ± SD. Data were acquired and analysed in a double‐blinded manner. The investigators who performed the analysis comprised a separate group from investigators who performed experiments, and the two separate groups worked without knowing the different treatments designed by the principle investigator. The unpaired Student’s *t*‐test was used to compare two groups with Shapiro–Wilk test for normality test. One‐way ANOVA was performed with Levene’s test for homogeneity of variance, followed by the Bonferroni post hoc test based on the comparison to be made and the statistical indication of each test. Mauchly’s sphericity test was used for sphericity test. For non‐parametric data, differences between groups were evaluated with non‐parametric Mann–Whitney U‐test, and categorical and binary variables were tested by the Fisher exact test. The exact sample size and the number of independent experiments performed, description of the samples and statistical analyses done were also specified in the figure legends. Statistical significance was accepted at *P* < 0.05. All graphs were generated using Prism V.7 (GraphPad), and all statistical tests were performed using SPSS V.21 (IBM). For detailed exact *P*‐value per figure panel, see Appendix Table S4.

### Ethics approval

Medical Ethics Committee of the First Affiliated Hospital of Sun Yat‐sen University.

## Author contributions

XL, SC, and ZH contributed equally to this work. HL conceived the ideas for experimental designs. XL, SC, and ZH conducted the majority of the experiments, analysed data, and prepared the manuscript. ZH, HW, and DC conducted sample collection and performed statistical analysis. JW, DC, ZL, and GD provided critical suggestions and instructions for the project and helped compose the manuscript. XL and SC provided μCT analysis. XL, SC, ZL, HC, and LL conducted the most animal experiments and performed analysis. KZ, ZZhe, and ZZha provided suggestions for the project. HL developed the concept, supervised the project, and conducted data analysis.

## Conflict of interest

The authors declare that they have no conflict of interest.

## Supporting information



Appendix

Expanded View Figures PDF

Review Process File

Source Data for Expanded View

## Data Availability

This study includes no data deposited in external repositories.

## References

[emmm202012109-bib-0001] Adarichev VA , Glant TT (2006) Experimental spondyloarthropathies: animal models of ankylosing spondylitis. Curr Rheumatol Rep 8: 267–274 16839505 10.1007/s11926-006-0007-5

[emmm202012109-bib-0002] Bardos T , Szabo Z , Czipri M , Vermes C , Tunyogi‐Csapo M , Urban RM , Mikecz K , Glant TT (2005) A longitudinal study on an autoimmune murine model of ankylosing spondylitis. Ann Rheum Dis 64: 981–987 15640265 10.1136/ard.2004.029710 PMC1755557

[emmm202012109-bib-0003] Berlo SE , Guichelaar T , Ten Brink CB , van Kooten PJ , Hauet‐Broeren F , Ludanyi K , van Eden W , Broeren CP , Glant TT (2006) Increased arthritis susceptibility in cartilage proteoglycan‐specific T cell receptor‐transgenic mice. Arthritis Rheum 54: 2423–2433 16869010 10.1002/art.22013

[emmm202012109-bib-0004] Bruijnen STG , Verweij NJF , van Duivenvoorde LM , Bravenboer N , Baeten DLP , van Denderen CJ , van der Horst‐Bruinsma IE , Voskuyl AE , Custers M , van de Ven PM *et al* (2018) Bone formation in ankylosing spondylitis during anti‐tumour necrosis factor therapy imaged by 18F‐fluoride positron emission tomography. Rheumatology 57: 770 29415219 10.1093/rheumatology/key034 PMC5888954

[emmm202012109-bib-0005] Chang W , Tu C , Chen TH , Bikle D , Shoback D (2008) The extracellular calcium‐sensing receptor (CaSR) is a critical modulator of skeletal development. Sci Signal 1: ra1 18765830 10.1126/scisignal.1159945 PMC3538864

[emmm202012109-bib-0006] Cheng SX (2016) Calcium‐sensing receptor: a new target for therapy of diarrhea. World J Gastroenterol 22: 2711–2724 26973410 10.3748/wjg.v22.i9.2711 PMC4777994

[emmm202012109-bib-0007] Cheng Z , Li A , Tu CL , Maria CS , Szeto N , Herberger A , Chen TH , Song F , Wang J , Liu X *et al* (2020) Calcium‐sensing receptors in chondrocytes and osteoblasts are required for callus maturation and fracture healing in mice. J Bone Miner Res 35: 143–154 31498905 10.1002/jbmr.3864 PMC7700777

[emmm202012109-bib-0008] Deodhar A (2018) TNF inhibitors and structural damage in ankylosing spondylitis. Nat Rev Rheumatol 14: 5–6 29213122 10.1038/nrrheum.2017.197

[emmm202012109-bib-0009] Dvorak‐Ewell MM , Chen TH , Liang N , Garvey C , Liu B , Tu C , Chang W , Bikle DD , Shoback DM (2011) Osteoblast extracellular Ca^2+^ ‐sensing receptor regulates bone development, mineralization, and turnover. J Bone Miner Res 26: 2935–2947 21956637 10.1002/jbmr.520 PMC3222747

[emmm202012109-bib-0010] Godwin SL , Soltoff SP (2002) Calcium‐sensing receptor‐mediated activation of phospholipase C‐gamma 1 is downstream of phospholipase C‐beta and protein kinase C in MC3T3‐E1 osteoblasts. Bone 30: 559–566 11934646 10.1016/s8756-3282(01)00700-1

[emmm202012109-bib-0011] Goltzman D , Hendy GN (2015) The calcium‐sensing receptor in bone–mechanistic and therapeutic insights. Nat Rev Endocrinol 11: 298–307 25752283 10.1038/nrendo.2015.30

[emmm202012109-bib-0012] Gonzalez‐Chavez SA , Quinonez‐Flores CM , Pacheco‐Tena C (2016) Molecular mechanisms of bone formation in spondyloarthritis. Joint Bone Spine 83: 394–400 26838262 10.1016/j.jbspin.2015.07.008

[emmm202012109-bib-0013] Gonzalez‐Vazquez A , Planell JA , Engel E (2014) Extracellular calcium and CaSR drive osteoinduction in mesenchymal stromal cells. Acta Biomater 10: 2824–2833 24525034 10.1016/j.actbio.2014.02.004

[emmm202012109-bib-0014] Gowen M , Stroup GB , Dodds RA , James IE , Votta BJ , Smith BR , Bhatnagar PK , Lago AM , Callahan JF , DelMar EG *et al* (2000) Antagonizing the parathyroid calcium receptor stimulates parathyroid hormone secretion and bone formation in osteopenic rats. J Clin Invest 105: 1595–1604 10841518 10.1172/JCI9038 PMC300853

[emmm202012109-bib-0015] Hannan FM , Babinsky VN , Thakker RV (2016) Disorders of the calcium‐sensing receptor and partner proteins: insights into the molecular basis of calcium homeostasis. J Mol Endocrinol 57: R127–R142 27647839 10.1530/JME-16-0124 PMC5064759

[emmm202012109-bib-0016] Hannan FM , Kallay E , Chang W , Brandi ML , Thakker RV (2018a) The calcium‐sensing receptor in physiology and in calcitropic and noncalcitropic diseases. Nat Rev Endocrinol 15: 33–51 30443043 10.1038/s41574-018-0115-0 PMC6535143

[emmm202012109-bib-0017] Hanyecz A , Berlo SE , Szanto S , Broeren CP , Mikecz K , Glant TT (2004) Achievement of a synergistic adjuvant effect on arthritis induction by activation of innate immunity and forcing the immune response toward the Th1 phenotype. Arthritis Rheum 50: 1665–1676 15146438 10.1002/art.20180

[emmm202012109-bib-0018] Hendy GN , Canaff L (2016) Calcium‐sensing receptor, proinflammatory cytokines and calcium homeostasis. Semin Cell Dev Biol 49: 37–43 26612442 10.1016/j.semcdb.2015.11.006

[emmm202012109-bib-0019] Hibino N , Hamada Y , Sairyo K , Yukata K , Sano T , Yasui N (2007) Callus formation during healing of the repaired tendon‐bone junction. J Bone Joint Surg Br 89b: 1539–1544 10.1302/0301-620X.89B11.19847 17998198

[emmm202012109-bib-0020] Holmdahl R , Jansson L , Andersson M , Jonsson R (1992) Genetic, hormonal and behavioural influence on spontaneously developing arthritis in normal mice. Clin Exp Immunol 88: 467–472 1606732 10.1111/j.1365-2249.1992.tb06473.x PMC1554520

[emmm202012109-bib-0021] Jacques P , Lambrecht S , Verheugen E , Pauwels E , Kollias G , Armaka M , Verhoye M , Van der Linden A , Achten R , Lories RJ *et al* (2014) Proof of concept: enthesitis and new bone formation in spondyloarthritis are driven by mechanical strain and stromal cells. Ann Rheum Dis 73: 437–445 23921997 10.1136/annrheumdis-2013-203643

[emmm202012109-bib-0022] Jacques P , McGonagle D (2014) The role of mechanical stress in the pathogenesis of spondyloarthritis and how to combat it. Best Pract Res Clin Rheumatol 28: 703–710 25488779 10.1016/j.berh.2014.10.009

[emmm202012109-bib-0023] Klein GL , Castro SM , Garofalo RP (2016) The calcium‐sensing receptor as a mediator of inflammation. Semin Cell Dev Biol 49: 52–56 26303192 10.1016/j.semcdb.2015.08.006 PMC4761504

[emmm202012109-bib-0024] Kreiner DS , Shaffer WO , Baisden JL , Gilbert TJ , Summers JT , Toton JF , Hwang SW , Mendel RC , Reitman CA , North American Spine S (2013) An evidence‐based clinical guideline for the diagnosis and treatment of degenerative lumbar spinal stenosis (update). Spine J 13: 734–743 23830297 10.1016/j.spinee.2012.11.059

[emmm202012109-bib-0025] Lee JW , Park HA , Kwon OK , Park JW , Lee G , Lee HJ , Lee SJ , Oh SR , Ahn KS (2017) NPS 2143, a selective calcium‐sensing receptor antagonist inhibits lipopolysaccharide‐induced pulmonary inflammation. Mol Immunol 90: 150–157 28800474 10.1016/j.molimm.2017.07.012

[emmm202012109-bib-0026] Lenke LG , Betz RR , Harms J , Bridwell KH , Clements DH , Lowe TG , Blanke K (2001) Adolescent idiopathic scoliosis: a new classification to determine extent of spinal arthrodesis. J Bone Joint Surg Am 83: 1169–1181 11507125

[emmm202012109-bib-0027] Li X , Li Z , Wang J , Li Z , Cui H , Dai G , Chen S , Zhang M , Zheng Z , Zhan Z *et al* (2019) Wnt4 signaling mediates protective effects of melatonin on new bone formation in an inflammatory environment. FASEB J 33: 10126–10139 31216173 10.1096/fj.201900093RR

[emmm202012109-bib-0028] Li X , Wang JR , Zhan ZP , Li SB , Zheng ZM , Wang TP , Zhang KB , Pan HH , Li ZM , Zhang N *et al* (2018) Inflammation intensity‐dependent expression of osteoinductive Wnt proteins is critical for ectopic new bone formation in ankylosing spondylitis. Arthritis Rheumatol 70: 1056–1070 29481736 10.1002/art.40468

[emmm202012109-bib-0029] Lories RJ , Derese I , Luyten FP (2005) Modulation of bone morphogenetic protein signaling inhibits the onset and progression of ankylosing enthesitis. J Clin Invest 115: 1571–1579 15902307 10.1172/JCI23738 PMC1090472

[emmm202012109-bib-0030] Lories RJ , Dougados M (2012) Inflammation and ankylosis: still an enigmatic relationship in spondyloarthritis. Ann Rheum Dis 71: 317–318 22315213 10.1136/annrheumdis-2011-200842

[emmm202012109-bib-0031] Lories RJ , Haroon N (2017) Evolving concepts of new bone formation in axial spondyloarthritis: Insights from animal models and human studies. Best Pract Res Cl Rh 31: 877–886 10.1016/j.berh.2018.07.007 30509446

[emmm202012109-bib-0032] Lories RJ , Matthys P , de Vlam K , Derese I , Luyten FP (2004) Ankylosing enthesitis, dactylitis, and onychoperiostitis in male DBA/1 mice: a model of psoriatic arthritis. Ann Rheum Dis 63: 595–598 15082495 10.1136/ard.2003.013599 PMC1754976

[emmm202012109-bib-0033] Ma JX , Wang B , Ding CF , Li HS , Jiang XJ , Wang CY , Yu J , Chen WQ (2020) Couplet medicines of leech and centipede granules improves erectile dysfunction via inactivation of the CaSR/PLC/PKC signaling in streptozotocin‐induced diabetic rats. Biosci Rep 40:BSR20193845 31922200 10.1042/BSR20193845 PMC7000366

[emmm202012109-bib-0034] Maksymowych WP , Chiowchanwisawakit P , Clare T , Pedersen SJ , Ostergaard M , Lambert RGW (2009) Inflammatory lesions of the spine on magnetic resonance imaging predict the development of new syndesmophytes in ankylosing spondylitis. Arthritis Rheum‐Us 60: 93–102 10.1002/art.24132 19116919

[emmm202012109-bib-0035] Matthys P , Lories RJ , De Klerck B , Heremans H , Luyten FP , Billiau A (2003) Dependence on interferon‐gamma for the spontaneous occurrence of arthritis in DBA/1 mice. Arthritis Rheum 48: 2983–2988 14558106 10.1002/art.11273

[emmm202012109-bib-0036] McClure J (1983) The effect of diphosphonates on heterotopic ossification in regenerating Achilles tendon of the mouse. J Pathol 139: 419–430 6220140 10.1002/path.1711390403

[emmm202012109-bib-0037] McGonagle DG , McInnes IB , Kirkham BW , Sherlock J , Moots R (2019) The role of IL‐17A in axial spondyloarthritis and psoriatic arthritis: recent advances and controversies. Ann Rheum Dis 78: 1167–1178 31278139 10.1136/annrheumdis-2019-215356 PMC6788885

[emmm202012109-bib-0038] Mikecz K , Glant TT , Poole AR (1987) Immunity to cartilage proteoglycans in BALB/c mice with progressive polyarthritis and ankylosing spondylitis induced by injection of human cartilage proteoglycan. Arthritis Rheum 30: 306–318 3566822 10.1002/art.1780300310

[emmm202012109-bib-0039] Milara J , Mata M , Serrano A , Peiro T , Morcillo EJ , Cortijo J (2010) Extracellular calcium‐sensing receptor mediates human bronchial epithelial wound repair. Biochem Pharmacol 80: 236–246 20381461 10.1016/j.bcp.2010.03.035

[emmm202012109-bib-0040] Mizumachi H , Yoshida S , Tomokiyo A , Hasegawa D , Hamano S , Yuda A , Sugii H , Serita S , Mitarai H , Koori K *et al* (2017) Calcium‐sensing receptor‐ERK signaling promotes odontoblastic differentiation of human dental pulp cells. Bone 101: 191–201 28506888 10.1016/j.bone.2017.05.012

[emmm202012109-bib-0041] Moenning A , Jager R , Egert A , Kress W , Wardelmann E , Schorle H (2009) Sustained platelet‐derived growth factor receptor alpha signaling in osteoblasts results in craniosynostosis by overactivating the phospholipase C‐gamma pathway. Mol Cell Biol 29: 881–891 19047372 10.1128/MCB.00885-08 PMC2630675

[emmm202012109-bib-0042] Molnar C , Scherer A , Baraliakos X , de Hooge M , Micheroli R , Exer P , Kissling RO , Tamborrini G , Wildi LM , Nissen MJ *et al* (2018) TNF blockers inhibit spinal radiographic progression in ankylosing spondylitis by reducing disease activity: results from the Swiss Clinical Quality Management cohort. Ann Rheum Dis 77: 63–69 28939631 10.1136/annrheumdis-2017-211544 PMC5754737

[emmm202012109-bib-0043] Pedersen SJ , Chiowchanwisawakit P , Lambert RGW , Ostergaard M , Maksymowych WP (2011) Resolution of inflammation following treatment of ankylosing spondylitis is associated with new bone formation. J Rheumatol 38: 1349–1354 21459937 10.3899/jrheum.100925

[emmm202012109-bib-0044] Ramiro S , Landewe R , van Tubergen A , Boonen A , Stolwijk C , van den Bosch F , Dougados M , van der Heijde D (2015) Lifestyle factors may modify the effect of disease activity on radiographic progression in patients with ankylosing spondylitis: a longitudinal analysis. RMD Open 1: e000153. 26535153 10.1136/rmdopen-2015-000153 PMC4623363

[emmm202012109-bib-0045] Raspanti M , Strocchi R , De Pasquale V , Martini D , Montanari C , Ruggeri A (1996) Structure and ultrastructure of the bone/ligament junction. Ital J Anat Embryol 101: 97–105 8997905

[emmm202012109-bib-0046] Ruiz‐Heiland G , Horn A , Zerr P , Hofstetter W , Baum W , Stock M , Distler JH , Nimmerjahn F , Schett G , Zwerina J (2012) Blockade of the hedgehog pathway inhibits osteophyte formation in arthritis. Ann Rheum Dis 71: 400–407 22233602 10.1136/ard.2010.148262

[emmm202012109-bib-0047] Sarem M , Heizmann M , Barbero A , Martin I , Shastri VP (2018) Hyperstimulation of CaSR in human MSCs by biomimetic apatite inhibits endochondral ossification via temporal down‐regulation of PTH1R. Proc Natl Acad Sci USA 115: E6135–E6144 29915064 10.1073/pnas.1805159115 PMC6142224

[emmm202012109-bib-0048] Schett G , Lories RJ , D'Agostino MA , Elewaut D , Kirkham B , Soriano ER , McGonagle D (2017) Enthesitis: from pathophysiology to treatment. Nat Rev Rheumatol 13: 731–741 29158573 10.1038/nrrheum.2017.188

[emmm202012109-bib-0049] Sciubba DM , Nelson C , Hsieh P , Gokaslan ZL , Ondra S , Bydon A (2008) Perioperative challenges in the surgical management of ankylosing spondylitis. Neurosurg Focus 24: E10 10.3171/FOC/2008/24/1/E10 18290736

[emmm202012109-bib-0050] Sherlock JP , Joyce‐Shaikh B , Turner SP , Chao CC , Sathe M , Grein J , Gorman DM , Bowman EP , McClanahan TK , Yearley JH *et al* (2012) IL‐23 induces spondyloarthropathy by acting on ROR‐gammat+ CD3+CD4‐CD8‐ entheseal resident T cells. Nat Med 18: 1069–1076 22772566 10.1038/nm.2817

[emmm202012109-bib-0051] Sieper J , Poddubnyy D (2016) New evidence on the management of spondyloarthritis. Nat Rev Rheumatol 12: 282–295 27052489 10.1038/nrrheum.2016.42

[emmm202012109-bib-0052] Sieper J , Poddubnyy D (2017) Axial spondyloarthritis. Lancet 390: 73–84 28110981 10.1016/S0140-6736(16)31591-4

[emmm202012109-bib-0053] Sims AM , Timms AE , Bruges‐Armas J , Burgos‐Vargas R , Chou CT , Doan T , Dowling A , Fialho RN , Gergely P , Gladman DD *et al* (2008) Prospective meta‐analysis of interleukin 1 gene complex polymorphisms confirms associations with ankylosing spondylitis. Ann Rheum Dis 67: 1305–1309 18063673 10.1136/ard.2007.081364

[emmm202012109-bib-0054] Song IH , Althoff C , Haibel H , Listing J , Weiss A , Freundlich B , Rudwaleit M , Sieper J (2012) Description of distribution of active inflammatory lesions on magnetic resonance imaging of the sacroiliac joints and the spine in patients with early axial spondyloarthritis ‐ analysis of the esther trial baseline data. Arthritis Rheum‐Us 64: S249

[emmm202012109-bib-0055] Szabo Z , Szanto S , Vegvari A , Szekanecz Z , Mikecz K , Glant TT (2005) Genetic control of experimental spondylarthropathy. Arthritis Rheum 52: 2452–2460 16059927 10.1002/art.21193

[emmm202012109-bib-0056] Taurog JD , Chhabra A , Colbert RA (2016) Ankylosing spondylitis and axial spondyloarthritis. New Engl J Med 374: 2563–2574 27355535 10.1056/NEJMra1406182

[emmm202012109-bib-0057] Tseng HW , Glant TT , Brown MA , Kenna TJ , Thomas GP , Pettit AR (2017) Early anti‐inflammatory intervention ameliorates axial disease in the proteoglycan‐induced spondylitis mouse model of ankylosing spondylitis. BMC Musculoskelet Disord 18: 228 28558827 10.1186/s12891-017-1600-7 PMC5450150

[emmm202012109-bib-0058] Tseng HW , Pitt ME , Glant TT , McRae AF , Kenna TJ , Brown MA , Pettit AR , Thomas GP (2016) Inflammation‐driven bone formation in a mouse model of ankylosing spondylitis: sequential not parallel processes. Arthritis Res Ther 18: 35 26831337 10.1186/s13075-015-0805-0 PMC4734853

[emmm202012109-bib-0059] van der Heijde D , Machado P , Braun J , Hermann KGA , Baraliakos X , Hsu B , Baker D , Landewe R (2012) MRI inflammation at the vertebral unit only marginally predicts new syndesmophyte formation: a multilevel analysis in patients with ankylosing spondylitis. Ann Rheum Dis 71: 369–373 21979001 10.1136/annrheumdis-2011-200208

[emmm202012109-bib-0060] van der Paardt M , Crusius JB , Garcia‐Gonzalez MA , Baudoin P , Kostense PJ , Alizadeh BZ , Dijkmans BA , Pena AS , van der Horst‐Bruinsma IE (2002) Interleukin‐1beta and interleukin‐1 receptor antagonist gene polymorphisms in ankylosing spondylitis. Rheumatology 41: 1419–1423 12468823 10.1093/rheumatology/41.12.1419

[emmm202012109-bib-0061] van Tok MN , van Duivenvoorde LM , Kramer I , Ingold P , Pfister S , Roth L , Blijdorp IC , van de Sande MGH , Taurog JD , Kolbinger F *et al* (2019) Interleukin‐17A inhibition diminishes inflammation and new bone formation in experimental spondyloarthritis. Arthritis Rheumatol 71: 612–625 30390386 10.1002/art.40770

[emmm202012109-bib-0062] Vieira‐Sousa E , van Duivenvoorde LM , Fonseca JE , Lories RJ , Baeten DL (2015) Animal models as a tool to dissect pivotal pathways driving spondyloarthritis. Arthritis Rheumatol 67: 2813–2827 26215401 10.1002/art.39282

[emmm202012109-bib-0063] Wang X , Li FF , Xie L , Crane J , Zhen GH , Mishina Y , Deng RX , Gao B , Chen H , Liu S *et al* (2018a) Inhibition of overactive TGF‐beta attenuates progression of heterotopic ossification in mice. Nat Commun 9: 551 29416028 10.1038/s41467-018-02988-5 PMC5803194

[emmm202012109-bib-0064] Wang X , Xie L , Crane J , Zhen G , Li F , Yang P , Gao M , Deng R , Wang Y , Jia X *et al* (2018b) Aberrant TGF‐beta activation in bone tendon insertion induces enthesopathy‐like disease. J Clin Invest 128: 846–860 29355842 10.1172/JCI96186 PMC5785263

[emmm202012109-bib-0065] Wu QY , Sun MR , Wu CL , Li Y , Du JJ , Zeng JY , Bi HL , Sun YH (2015) Activation of calcium‐sensing receptor increases TRPC3/6 expression in T lymphocyte in sepsis. Mol Immunol 64: 18–25 25467798 10.1016/j.molimm.2014.10.018

[emmm202012109-bib-0066] Xie ZY , Wang P , Li YX , Deng W , Zhang X , Su HJ , Li D , Wu YF , Shen HY (2016) Imbalance between bone morphogenetic protein 2 and noggin induces abnormal osteogenic differentiation of mesenchymal stem cells in ankylosing spondylitis. Arthritis Rheumatol 68: 430–440 26413886 10.1002/art.39433

[emmm202012109-bib-0067] Yamaguchi T , Chattopadhyay N , Kifor O , Sanders JL , Brown EM (2000) Activation of p42/44 and p38 mitogen‐activated protein kinases by extracellular calcium‐sensing receptor agonists induces mitogenic responses in the mouse osteoblastic MC3T3‐E1 cell line. Biochem Bioph Res Co 279: 363–368 10.1006/bbrc.2000.3955 11118293

[emmm202012109-bib-0068] Yarova PL , Stewart AL , Sathish V , Britt RD , Thompson MA , Lowe APP , Freeman M , Aravamudan B , Kita H , Brennan SC *et al* (2015) Calcium‐sensing receptor antagonists abrogate airway hyperresponsiveness and inflammation in allergic asthma. Sci Transl Med 7: 284 10.1126/scitranslmed.aaa0282 PMC4725057 25904744

[emmm202012109-bib-0069] Zhang W , Sun R , Zhong H , Tang N , Liu Y , Zhao Y , Zhang T , He F (2019) CaSR participates in the regulation of vascular tension in the mesentery of hypertensive rats via the PLCIP3/ACV/cAMP/RAS pathway. Mol Med Rep 20: 4433–4448 31485595 10.3892/mmr.2019.10620 PMC6797953

