# Supplementary material for: Aberrant upregulation of CaSR promotes pathological new bone formation in ankylosing spondylitis
Source: EMBO Mol Med. 2020 Dec 1;12(12):e12109. doi: 10.15252/emmm.202012109 (PMC7721361; doi:10.15252/emmm.202012109)
Supplement: Supplementary file 1 — Appendix [file EMMM-12-e12109-s001.docx]

**Appendix**

Appensix Table S1. Demographic and clinical data of patients

Appensix Table S2. Primers for RT-qPCR analysis of gene expression

Appensix Table S3. siRNA sequences for RNA interference

Appendix Table S4. Exact P values

**Appensix Table S1**

**Appensix Table S1. Demographic and clinical data of patients**

|  | non-AS  n=24 | AS  n=18 |
| --- | --- | --- |
| Sample | Spinal tissues | Spinal tissues |
| Age | 39.3 ± 5.8 | 34.1 ± 9.1 |
| Sex(M/F) | 19/5 | 14/4 |
| Disease period | / | 9.3 ± 5.2 |
| BASDAI | / | 4.8 ± 1.3 |
| BASFI | / | 5.8 ± 0.9 |

**Appensix Table S1.** Summary of Demographic and clinical data of patients.

**Appensix Table S2**

**Appensix Table S2. Primers for QRT-PCR Analysis of Gene Expression**

| Primer | 5’ Forward 3’ | 5’ Reverse 3’ |
| --- | --- | --- |
| Actin | CGTGCGTGACATCAAAGAGAAG | CGTTGCCAATAGTGATGACCTG |
| CaSR | AGCAGGTGACCTTCGATGAGT | ACTTCCTTGAACACAATGGAGC |
| Runx2 | GACTGTGGTTACCGTCATGGC | ACTTGGTTTTTCATAACAGCGGA |
| Osx | CCTCTTGAGAGGAGACGGGA | TGTACCACGAGCCATAGGGA |
| ALP | CCAACTCTTTTGTGCCAGAGA | GGCTACATTGGTGTTGAGCTTTT |
| OCN | TGAGTCTGACAAAGCCTTC | CTGCTGTGACATCCATACTTG |
| p65 | GAGACCTGGAGCAAGCCATT | GCCTGGTCCCGTGAAATACA |
| Stat3 | TCGCTCACGTTTGACATGGA | TCTAACAACCAACCCCGAGC |
| Col2a1 | GGGAATGTCCTCTGCGATGAC | GAAGGGGATCTCGGGGTTG |
| Sox9 | GAGCCGGATCTGAAGAGGGA | GCTTGACGTGTGGCTTGTTC |
| TNFα | CCCTCACACTCAGATCATCTTCT | GCTACGACGTGGGCTACAG |
| IL-1β | GCAACTGTTCCTGAACTCAACT | ATCTTTTGGGGTCCGTCAACT |
| IL-17A | TTTAACTCCCTTGGCGCAAAA | CTTTCCCTCCGCATTGACAC |
| IL-22 | ATGAGTTTTTCCCTTATGGGGAC | GCTGGAAGTTGGACACCTCAA |
| IL-23 | ATGCTGGATTGCAGAGCAGTA | ACGGGGCACATTATTTTTAGTCT |

**Appensix Table S2.** Summary of Primers for QRT-PCR Analysis of Gene Expression.

**Appensix Table S3**

**Appensix Table S3. siRNA sequences for RNA interference**

| Gene | Sense | Antisense |
| --- | --- | --- |
| Control | UAACGACGCGACGACGUAATT | UUACGUCGUCGCGUCGUUATT |
| p65 | GCAUGCGAUUCCGCUAUAATT | UUAUAGCGGAAUCGCAUGCTT |
| Stat3 | AAAUGAAGGUGGUGGAGAAUUTT | UUCUCCACCACCUUCAUUUUUTT |
| CaSR | GAGUGCAUCAGGUAUAACUTT | AGUUAUACCUCAGGCACUCTT |

**Appensix Table S3.** Summary of siRNA sequences for RNA interference.

**Appendix Table S4.**

**Appendix Table S4. Exact P-values**

| Figure 1 | P value |
| --- | --- |
| 1C CaSR^+^ cell number (AS vs Ctrl) | <0.0001 |
| 1C Runx2^+^ cell number  (AS vs Ctrl) | <0.0001 |
| 1D CaSR mRNA level  (AS vs Ctrl) | 0.0003 |
| 1G CaSR^+^ cell (%)  (AS vs Ctrl) | <0.0001 |
| 1G CaSR^+^ cell number  (AS vs Ctrl) | <0.0001 |
| 1G Runx2^+^ cell number  (AS vs Ctrl) | <0.0001 |
| 1G CaSR^+^ Runx2^+^ cell number (AS vs Ctrl) | <0.0001 |
| 1I CaSR^+^ cell (%)  (AS vs Ctrl) | <0.0001 |
| 1I CaSR^+^ cell number  (AS vs Ctrl) | <0.0001 |
| 1I Runx2^+^ cell number  (AS vs Ctrl) | <0.0001 |
| 1I CaSR^+^ Runx2^+^ cell number (AS vs Ctrl) | <0.0001 |

| (24W vs 16W) | 0.0011 |
| --- | --- |
| 2F CaSR^+^ Runx2^+^ cell |  |
| (16W vs 8W) | <0.0001 |
| (24W vs 8W) | <0.0001 |
| (24 vs 16W) | <0.0001 |
| 2H CaSR^+^ cell (%) |  |
| (30W vs 8W) | <0.0001 |
| 2H CaSR^+^ cell number |  |
| (30W vs 8W) | <0.0001 |
| 2H OCN^+^ cell number |  |
| (30W vs 8W) | <0.0001 |
| 2H CaSR^+^ OCN^+^ cell |  |
| (30W vs 8W) | <0.0001 |

| Figure 3 | Pvalue |
| --- | --- |
| 3B Incidence of Ankle enthesophyte | 0.001 |
| 3C BV |  |
| (16W vs 8W) | <0.0001 |
| (20W vs 16W) | <0.0001 |
| 3D CaSR mRNA level |  |
| (20W vs 8W) | <0.0001 |
| 3F CaSR^+^ cell (%) |  |
| (16W vs 8W) | <0.0001 |
| (20W vs 16W) | <0.0001 |
| 3F CaSR^+^ cell number |  |
| (16W vs 8W) | <0.0001 |
| (20W vs 16W) | <0.0001 |
| 3F Runx2^+^ cell number |  |
| (16W vs 8W) | <0.0001 |
| (20W vs 16W) | <0.0001 |
| 3F CaSR^+^ Runx2^+^ cell |  |
| (16W vs 8W) | <0.0001 |
| (20W vs 16W) | <0.0001 |
| 3H CaSR^+^ cell (%) | <0.0001 |
| 3H CaSR^+^ cell number | <0.0001 |
| 3H OCN^+^ cell number | <0.0001 |
| 3H CaSR^+^ OCN^+^ cell | <0.0001 |

| Figure 2 | P value |
| --- | --- |
| 2B Incidence of Spinal Ankylosis | 0.033 |
| 2C BV (16W vs 8W) | 0.0015 |
| 2C BV (24W vs 16W) | 0.0005 |
| 2D CaSR mRNA level(24W vs 8W) | 0.0004 |
| 2F CaSR^+^ cell (%) |  |
| (16W vs 8W) | <0.0001 |
| (24W vs 16W) | <0.0001 |
| 2F CaSR^+^ cell number |  |
| (16W vs 8W) | 0.0001 |
| (24W vs 16W) | <0.0001 |
| 2F Runx2^+^ cell number |  |
| (16W vs 8W) | <0.0001 |

| Fugure 4 | P value |
| --- | --- |
| 4C Incidence of Spinal Ankylosis | 0.032 |
| 4D BV |  |
| (SR vs OS) | <0.0001 |
| (NPS 24W vs Ctrl 24W) | <0.0001 |
| 4F Incidence of Ankle enthesophyte | 0.0110 |
| 4G BV |  |
| (20W vs 8W) | <0.0001 |
| (NPS 20W vs Ctrl 20W) | <0.0001 |
| 4K Area of UF |  |
| (8W vs Sham) | <0.0001 |
| (NPS 8W vs Ctrl 8W) | 0.001 |
| 4L Area of CF |  |
| (8W vs Sham) | <0.0001 |
| (NPS 8W vs Ctrl 8W) | 0.0008 |

| (U73122 vs SR) | 0.0003 |
| --- | --- |
| 5F mRNA level |  |
| Runx2 |  |
| (OS vs NT) | 0.0089 |
| (SR vs OS) | <0.0001 |
| (U73122 vs SR) | <0.0001 |
| Osx |  |
| (OS vs NT) | 0.0202 |
| (SR vs OS) | <0.0001 |
| (U73122 vs SR) | <0.0001 |
| ALP |  |
| (OS vs NT) | <0.0001 |
| (SR vs OS) | <0.0001 |
| (U73122 vs SR) | <0.0001 |
| OCN |  |
| (OS vs NT) | 0.0006 |
| (SR vs OS) | <0.0001 |
| (U73122 vs SR) | <0.0001 |
| 5I |  |
| CaSR^+^ cell |  |
| (24W vs 8W) | <0.0001 |
| (NPS vs Ctrl) | >0.9999 |
| p-PLCγ^+^ cell |  |
| (24W vs 8W) | <0.0001 |
| (NPS vs Ctrl) | <0.0001 |
| CaSR^+^ p-PLCγ^+^ cell |  |
| (24W vs 8W) | <0.0001 |
| (NPS vs Ctrl) | <0.0001 |
| 5J |  |
| CaSR^+^ cell |  |
| (24W vs 8W) | <0.0001 |
| (NPS vs Ctrl) | >0.9999 |
| p-PLCγ^+^ cell |  |
| (24W vs 8W) | <0.0001 |
| (NPS vs Ctrl) | 0.0001 |
| CaSR^+^ p-PLCγ^+^ cell |  |
| (24W vs 8W) | <0.0001 |
| (NPS vs Ctrl) | <0.0001 |
| 5K |  |
| CaSR^+^ cell |  |
| (24W vs 8W) | <0.0001 |
| (NPS vs Ctrl) | <0.0001 |

| Figure 5 | P value |
| --- | --- |
| 5B Alizarin Red |  |
| (OS vs NT) | <0.0001 |
| (DMSO vs OS) | >0.9999 |
| (SR vs OS) | <0.0001 |
| (NPS vs OS) | <0.0001 |
| 5C mRNA level |  |
| Runx2,Osx |  |
| (OS vs NT) | <0.0001 |
| (DMSO vs OS) | >0.9999 |
| (SR vs OS) | <0.0001 |
| (NPS vs OS) | <0.0001 |
| ALP |  |
| (OS vs NT) | <0.0001 |
| (DMSO vs OS) | >0.9999 |
| (SR vs OS) | <0.0001 |
| OCN |  |
| (OS vs NT) | <0.0001 |
| (DMSO vs OS) | >0.9999 |
| (SR vs OS) | 0.0002 |
| (NPS vs OS) | 0.0014 |
| 5E Alizarin Red |  |
| (OS vs NT) | 0.0147 |
| (SR vs OS) | <0.0001 |

| p-PLCγ^+^ cell |  |
| --- | --- |
| (24W vs 8W) | <0.0001 |
| (NPS vs Ctrl) | <0.0001 |
| CaSR^+^ p-PLCγ^+^ cell |  |
| (24W vs 8W) | <0.0001 |
| (NPS vs Ctrl) | <0.0001 |
| 5N Area of UF | <0.0001 |
| 5N Area of CF | <0.0001 |
| (sip65 vs IL-17A) | <0.0001 |

| 6C CaSR mRNA |  |
| --- | --- |
| 24h |  |
| 1ng/ml | >0.9999 |
| 10ng/ml | 0.0062 |
| 50ng/ml | >0.9999 |
| 48h, 72h |  |
| 1ng/ml | <0.0001 |
| 10ng/ml | <0.0001 |
| 50ng/ml | <0.0001 |
| 6C CaSR mRNA |  |
| (IL-17A vs NT) | <0.0001 |
| (siCtrl vs IL-17A) | >0.9999 |
| (sip65 vs IL-17A) | <0.0001 |
| 6C CaSR mRNA |  |
| (IL-17A vs NT) | <0.0001 |
| (siCtrl vs IL-17A) | >0.9999 |
| (siStat3 vs IL-17A) | 0.0123 |
| 6D CaSR mRNA |  |
| 24h, 48h, 72h |  |
| 1ng/ml | >0.9999 |
| 10ng/ml | <0.0001 |
| 50ng/ml | <0.0001 |
| 6D CaSR mRNA |  |
| (IL-22 vs NT) | <0.0001 |
| (siCtrl vs IL-22) | >0.9999 |
| (sip65 vs IL-22) | 0.7481 |
| 6D CaSR mRNA |  |
| (IL-22 vs NT) | <0.0001 |
| (siCtrl vs IL-22) | 0.1591 |
| (siStat3 vs IL-22) | <0.0001 |
| 6E CaSR mRNA |  |
| 24h |  |
| 1ng/ml | <0.0001 |
| 10ng/ml | >0.9999 |
| 50ng/ml | <0.0001 |
| 48h |  |
| 1ng/ml | 0.3049 |
| 10ng/ml | 0.0006 |
| 50ng/ml | <0.0001 |
| 72h |  |
| 1ng/ml | <0.0001 |
| 10ng/ml | <0.0001 |
| 50ng/ml | <0.0001 |

| Figure 6 | P value |
| --- | --- |
| 6A CaSR mRNA |  |
| 24h, 48h, 72h |  |
| 1ng/ml | >0.9999 |
| 10ng/ml | <0.0001 |
| 50ng/ml | <0.0001 |
| 6A CaSR mRNA |  |
| (IL-1β vs NT) | <0.0001 |
| (siCtrl vs IL-1β) | 0.6457 |
| (sip65 vs IL-1β) | <0.0001 |
| 6A CaSR mRNA |  |
| (IL-1β vs NT) | <0.0001 |
| (siCtrl vs IL-1β) | >0.9999 |
| (siStat3 vs IL-1β) | >0.9999 |
| 6B CaSR mRNA |  |
| 24h |  |
| 1ng/ml | >0.9999 |
| 10ng/ml | <0.0001 |
| 50ng/ml | <0.0001 |
| 48h,72h |  |
| 1ng/ml | <0.0001 |
| 10ng/ml | <0.0001 |
| 50ng/ml | <0.0001 |
| 6B CaSR mRNA |  |
| (TNFα vs NT) | <0.0001 |
| (siCtrl vs TNFα) | 0.2083 |
| (sip65 vs TNFα) | <0.0001 |
| 6B CaSR mRNA |  |
| (TNFα vs NT) | <0.0001 |
| (siCtrl vs TNFα) | >0.9999 |
| (siStat3 vs TNFα) | <0.0001 |

| 6E CaSR mRNA |  |
| --- | --- |
| (IL-23 vs NT) | <0.0001 |
| (siCtrl vs IL-23) | >0.9999 |
| (sip65 vs IL-23) | >0.9999 |
| 6E CaSR mRNA |  |
| (IL-23 vs NT) | <0.0001 |
| (siCtrl vs IL-23) | 0.9959 |
| (siStat3 vs IL-23) | <0.0001 |
|  |  |
| Figure 7 | P value |
| 7A CaSR^+^p-p65^+^ cell |  |
| (16W vs 8W) | <0.0001 |
| (24W vs 16W) | 0.0002 |
| 7A CaSR^+^p-Stat3^+^ cell |  |
| (16W vs 8W) | <0.0001 |
| (24W vs 16W) | 0.0014 |
| 7B CaSR^+^p-p65^+^ cell |  |
| (16W vs 8W) | <0.0001 |
| (20W vs 16W) | <0.0001 |
| 7B CaSR^+^p-Stat3^+^ cell |  |
| (16W vs 8W) | <0.0001 |
| (20W vs 16W) | <0.0001 |
| 7C CaSR^+^p-p65^+^ cel | <0.0001 |
| 7C CaSR^+^p-Stat3^+^ cell | <0.0001 |
|  |  |
| Figure EV1 | P value |
| EV1C Area of UF |  |
| (4W vs Sham) | <0.0001 |
| (8W vs 4W) | <0.0001 |
| EV1D Area of CF |  |
| (4W vs Sham) | 0.0009 |
| (8W vs 4W) | 0.0009 |
| EV1E CaSR mRNA | 0.0011 |
| EV1G CaSR^+^ cell(%) |  |
| (4W vs Sham) | 0.0002 |
| (8W vs 4W) | <0.0001 |
| EV1G CaSR^+^ cell number |  |
| (4W vs Sham) | <0.0001 |
| (8W vs 4W) | 0.0005 |
| EV1G Runx2^+^ cell number |  |
| (4W vs Sham) | <0.0001 |

| (8W vs 4W) | <0.0001 |
| --- | --- |
| EV1G CaSR^+^Runx2^+^ cell |  |
| (4W vs Sham) | <0.0001 |
| (8W vs 4W) | <0.0001 |
| EV1I CaSR^+^ cell(%) |  |
| (4W vs Sham) | 0.0004 |
| (8W vs 4W) | <0.0001 |
| EV1I CaSR^+^ cell number |  |
| (4W vs Sham) | <0.0001 |
| (8W vs 4W) | <0.0001 |
| EV1I OCN^+^ cell number |  |
| (4W vs Sham) | 0.0274 |
| (8W vs 4W) | <0.0001 |
| EV1I CaSR^+^OCN^+^ cell |  |
| (4W vs Sham) | <0.0001 |
| (8W vs 4W) | <0.0001 |
| EV1G CaSR^+^Runx2^+^ cell |  |
| (4W vs Sham) | <0.0001 |
| (8W vs 4W) | <0.0001 |
| EV1I CaSR^+^ cell(%) |  |
| (4W vs Sham) | 0.0004 |
| (8W vs 4W) | <0.0001 |
| EV1I CaSR^+^ cell number |  |
| (4W vs Sham) | <0.0001 |
| (8W vs 4W) | <0.0001 |
| EV1I OCN^+^ cell number |  |
| (4W vs Sham) | 0.0274 |
| (8W vs 4W) | <0.0001 |
| EV1I CaSR^+^OCN^+^ cell |  |
| (4W vs Sham) | <0.0001 |
| (8W vs 4W) | <0.0001 |
|  |  |
| Figure EV2 | P value |
| EV2A | 0.0625 |
| EV2B | 0.778 |
|  |  |
| Figure EV3 | P value |
| EV3B Sox9, Col2a1 |  |
| (SR vs Ctrl) | >0.9999 |
| (NPS vs Ctrl) | <0.0001 |
| EV3C |  |
| (IL-1β, IL-17A, IL-22 | >0.9999 |

| and IL-23 vs Ctrl) |  |
| --- | --- |
| (TNFα vs Ctrl) | 0.1800 |
|  |  |
| Figure EV4 | P value |
| EV4A |  |
| (IL-1β, TNFα,IL-17A, | <0.0001 |
| IL-22 and IL-23 16W vs 8W) |  |
| EV4B |  |
| (IL-1β, TNFα,IL-17A,and | <0.0001 |
| IL-22 and IL-23 16W vs 8W) |  |
| EV4C |  |
| (IL-1β, TNFα, IL-22) | <0.0001 |
| (IL-17A 2W vs Sham) | 0.0002 |
| (IL-23 2W vs Sham) | 0.0003 |
| EV4D Alizarin Red |  |
| (OS vs NT) | <0.0001 |
| (siCaSR vs siCtrl) | <0.0001 |
| EV4D mRNA level |  |
| Runx2 |  |
| (OS vs NT) | <0.0001 |
| (siCtrl vs OS) | 0.4298 |
| (siCaSR vs siCtrl) | <0.0001 |
| Osx, ALP, OCN |  |
| (OS vs NT) | <0.0001 |
| (siCtrl vs OS) | >0.9999 |
| (siCaSR vs siCtrl) | <0.0001 |
| EV4E Alizarin Red |  |
| (TNFα vs Ctrl) | <0.0001 |
| (siCtrl vs OS) | >0.9999 |
| (siCaSR vs siCtrl) | <0.0001 |
| EV4E mRNA level |  |
| Runx2, Osx, ALP, OCN |  |
| (TNFα vs Ctrl) | <0.0001 |
| (siCtrl vs OS) | >0.9999 |
| (siCaSR vs siCtrl) | <0.0001 |
| EV4F Alizarin Red |  |
| (Lv-CaSR vs EV) | <0.0001 |
| (sip65 vs siNC) | >0.9999 |
| (siStat3 vs siNC) | >0.9999 |

| EV4F mRNA level |  |
| --- | --- |
| Runx2 |  |
| (Lv-CaSR vs EV) | 0.0003 |
| (sip65 vs siNC) | >0.9999 |
| (siStat3 vs siNC) | >0.9999 |
| Osx, ALP |  |
| (Lv-CaSR vs EV) | <0.0001 |
| (sip65 vs siNC) | >0.9999 |
| (siStat3 vs siNC) | >0.9999 |
| OCN |  |
| (Lv-CaSR vs EV) | 0.0002 |
| (sip65 vs siNC) | >0.9999 |
| (siStat3 vs siNC) |  |
| >0.9999 |  |
| EV4G CaSR mRNA |  |
| (TNFα VS NT) | <0.0001 |
| (TNFα+IL-17A vs NT) | <0.0001 |
| (TNFα+IL-17A vs TNFα) | >0.9999 |
| (TNFα+IL-17AvsIL-17A) | <0.0001 |
| EV4H | <0.0001 |
| EV4I | <0.0001 |
| EV4J | <0.0001 |
|  |  |
| Figure EV5 | P value |
| EV5C CaSR mRNA |  |
| (siCtrl vs NT) | >0.9999 |
| (siCaSR vs siCtrl) | <0.0001 |
| EV5D p65 mRNA |  |
| (siCtrl vs NT) | >0.9999 |
| (sip65 vs siCtrl) | <0.0001 |
| EV5E Stat3 mRNA |  |
| (siCtrl vs NT) | >0.9999 |
| (siStat3 vs siCtrl) | <0.0001 |

**Appendix Table S4**. Exact P values for all comparisons in the manuscript.
