# Supplementary material for: Aberrant upregulation of CaSR promotes pathological new bone formation in ankylosing spondylitis
Source: EMBO Mol Med. 2020 Dec 1;12(12):e12109. doi: 10.15252/emmm.202012109 (PMC7721361; doi:10.15252/emmm.202012109)
Supplement: Supplementary file 2 — Expanded View Figures PDF [file EMMM-12-e12109-s002.pdf]

Expanded View Figures

Figure EV1. CaSR$^+$ osteoblasts mediate pathological new bone formation of SMTS model.

A µCT images of the PCT in SMTS model. Arrowhead shows bony projection. (2D).
B H&E and SOFG staining of Achilles tendon enthesis compartment in SMTS model. AT: Achilles tendon.
C Quantitative analysis of area of UF. $n = 5$, one-way ANOVA, Bonferroni *post hoc*.
D Quantitative analysis of area of CF. $n = 5$, one-way ANOVA, Bonferroni *post hoc*.
E RT–qPCR analysis of CaSR in SMTS model. $n = 3$, Student's *t*-test.
F Immunofluorescence analyses of Achilles tendon enthesis compartment in SMTS model.
G Quantitative analysis of CaSR$^+$, Runx2$^+$ and CaSR$^+$ Runx2$^+$ cells number and CaSR$^+$ cell percentage in Achilles tendon enthesis compartment. $n = 5$, one-way ANOVA, Bonferroni *post hoc*.
H Immunofluorescence analyses of Achilles tendon enthesis compartment in SMTS model.
I Quantitative analysis of CaSR$^+$, OCN$^+$ and CaSR$^+$ OCN$^+$ cells number in Achilles tendon enthesis compartment. $n = 5$, one-way ANOVA, Bonferroni *post hoc*.

Data information: Data shown as mean ± SD. *$P < 0.05$, **$P < 0.01$ compared between groups. Scale bar: 100 µm.

© 2020 The Authors

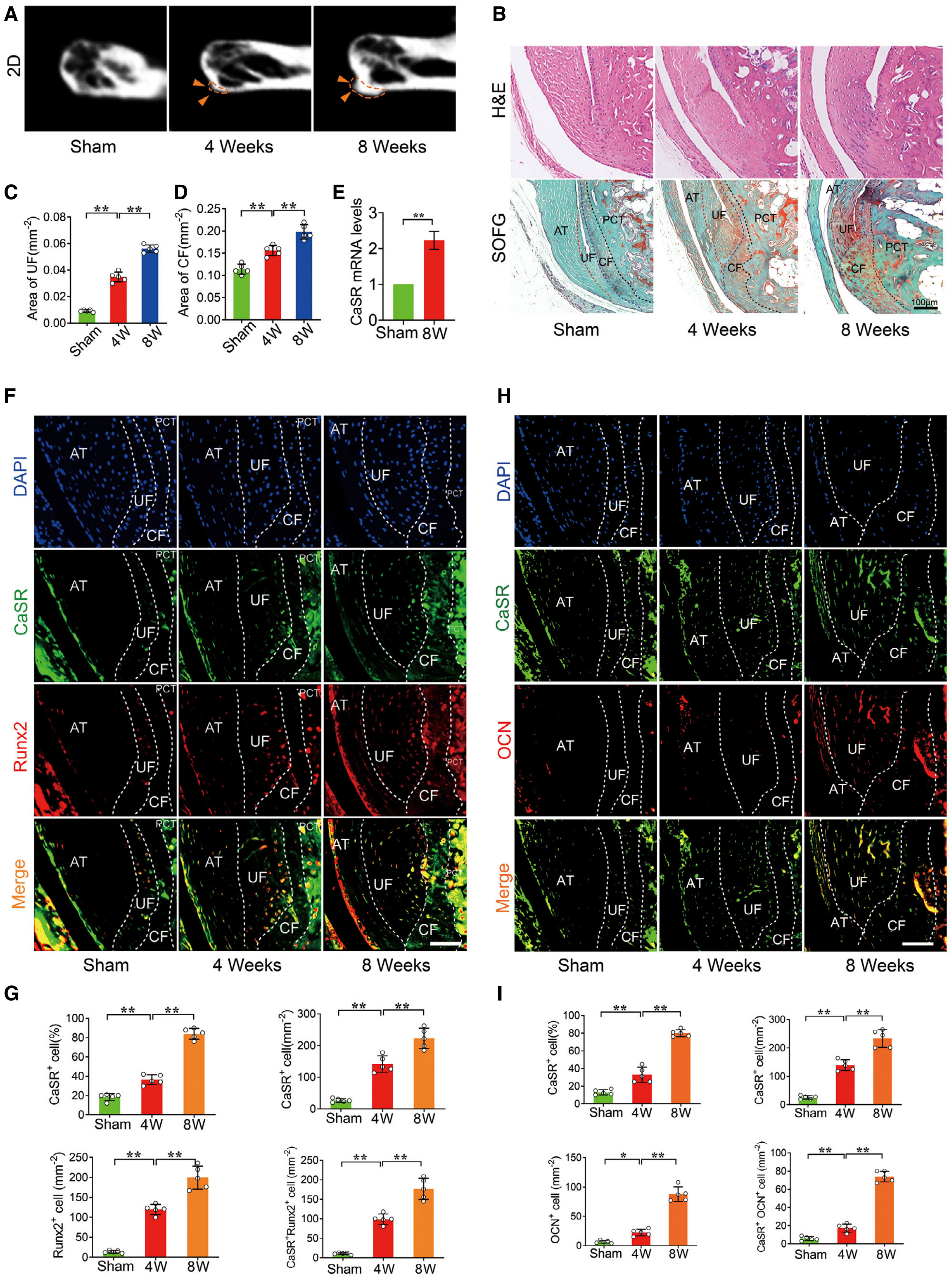

Figure EV1.

© 2020 The Authors

Figure EV2. NPS-2143 suppressed pathological new bone formation might be independent of PTH.

A Histological score of PGIS mice. $n = 5$ per group, Mann–Whitney *U*-test.
B Clinical severity scores of arthritis for DBA/1 mice. $n = 9$ per group. Repeated measures mixed models time × condition.
C Plasma PTH level in DBA/1, C57BL/6, and BALB/c mice after NPS-2143 treatment. $n = 3$ per group.

Data information: Data shown as mean ± SD. NS, not significant.

© 2020 The Authors

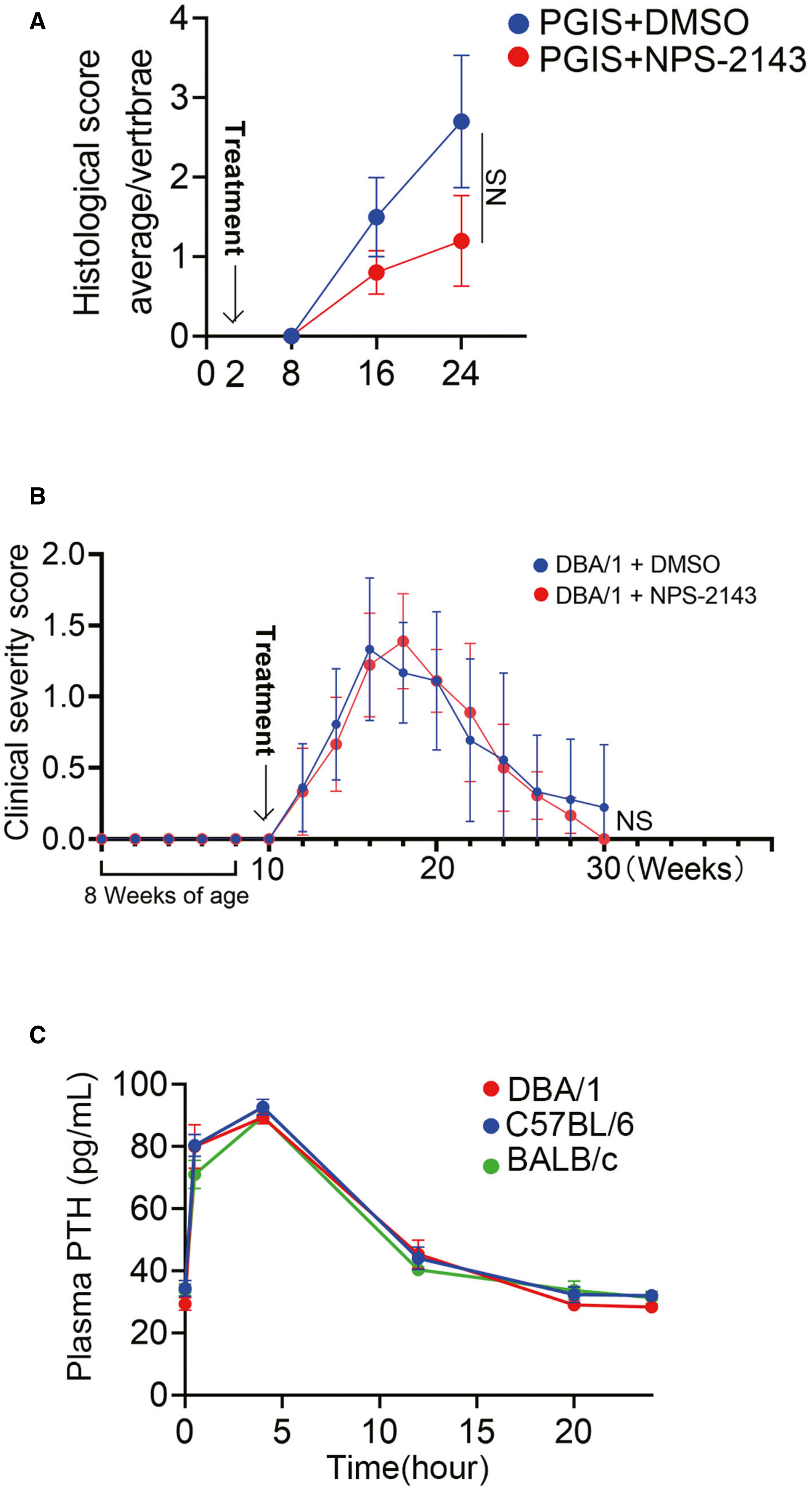

Figure EV2.

© 2020 The Authors

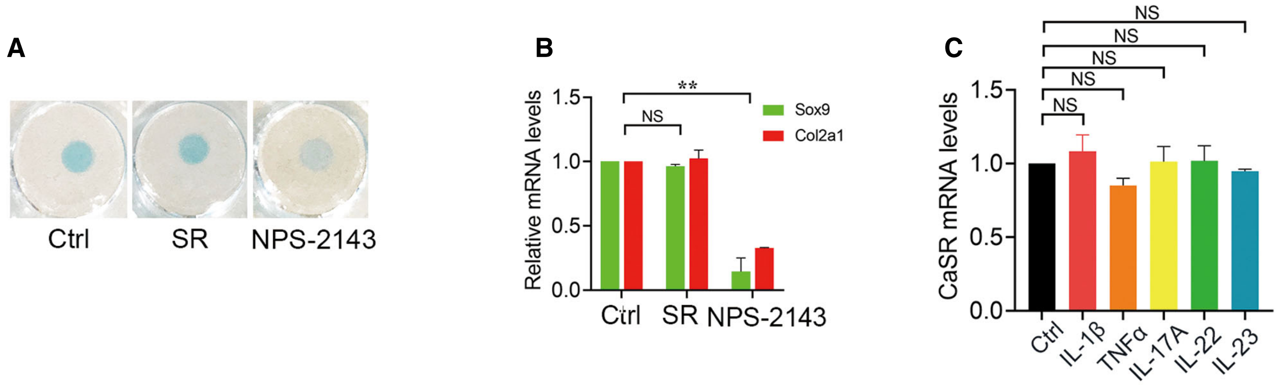

Figure EV3. The role of CaSR in chondrocytes.

A Alcian Blue staining of ATDC5 cells.
B RT–qPCR analysis of chondrogenic markers in ATDC5 cells. $n = 3$, one-way ANOVA, Bonferroni *post hoc*.
C RT–qPCR analysis of CaSR in ATDC5 cells. $n = 3$, one-way ANOVA, Bonferroni *post hoc*.

Data information: Data shown as mean ± SD. $^{**}P < 0.01$ compared between groups, NS: not significant, $P \geq 0.05$ compared between groups.

© 2020 The Authors

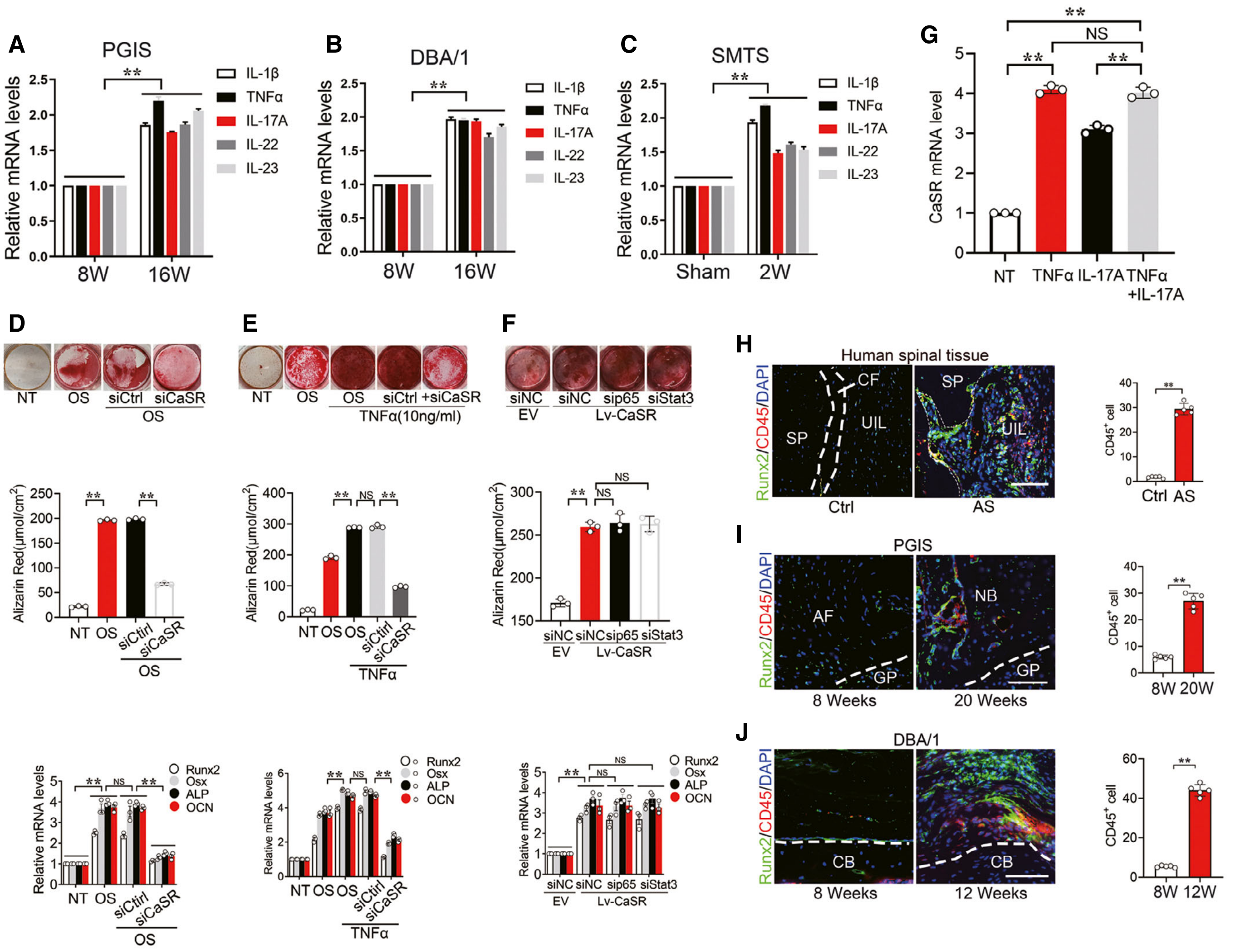

Figure EV4. The interaction between CaSR and inflammation in pathological new bone formation.

A–C RT–qPCR analysis of CaSR mRNA level of entheseal tissues in PGIS, DBA/1 and SMTS models. n = 5, Student's t-test.

D Alizarin Red staining of MC3T3-E1 cells. Quantification of Alizarin Red staining and RT–qPCR analysis of osteogenesis markers of MC3T3-E1 cells. n = 3, one-way ANOVA, Bonferroni *post hoc*.

E Alizarin Red staining of MC3T3-E1 cells. Quantification of Alizarin Red staining and RT–qPCR analysis of osteogenesis markers of MC3T3-E1 cells. n = 3, one-way ANOVA, Bonferroni *post hoc*.

F Alizarin Red staining of MC3T3-E1 cells. Quantification of Alizarin Red staining and RT–qPCR analysis of osteogenesis markers of MC3T3-E1 cells. n = 3, one-way ANOVA, Bonferroni *post hoc*.

G RT–qPCR analysis of mRNA level of CaSR in MC3T3-E1 cells. n = 3, one-way ANOVA, Bonferroni *post hoc*.

H Immunofluorescence analyses of $CD45^+$ and $Runx2^+$ cells at spinal entheseal site in human tissues. Quantitative analysis of $CD45^+$ cells. n = 5 per group, Student's t-test.

I Immunofluorescence analyses of $CD45^+$ and $Runx2^+$ cells at spinal entheseal site in PGIS model. Quantitative analysis of $CD45^+$ cells. n = 5 per group. Student's t-test.

J Immunofluorescence analyses of $CD45^+$ and $Runx2^+$ cells at ankle entheseal site in DBA/1 model. Quantitative analysis of $CD45^+$ cells. n = 5 per group. Student's t-test.

Data information: Data shown as mean ± SD. $^*P < 0.01$ or NS: $P \geq 0.05$ compared between groups.

© 2020 The Authors

Figure EV5. Functional verification of target-specific siRNAs and identification of hBMSCs.

A Representative CT scanning of AS patient. SP: spinous process, IL: interspinous ligament; SL: supraspinous ligament; CIL: calcified interspinous ligament; UIL: uncalcified interspinous ligament (collected tissues).

B hBMSCs exhibited positive staining for CD90, CD105 and CD73 but were negative for CD45 and CD14.

C–E RT–qPCR and Western blot analyses of expression of Stat3, p65 and CaSR for functional verification of target-specific siRNAs. $n = 3$, one-way ANOVA, Bonferroni *post hoc*.

Data information: Data shown as mean ± SD. $^{**}P < 0.01$ compare between groups. NS: not significant.

© 2020 The Authors

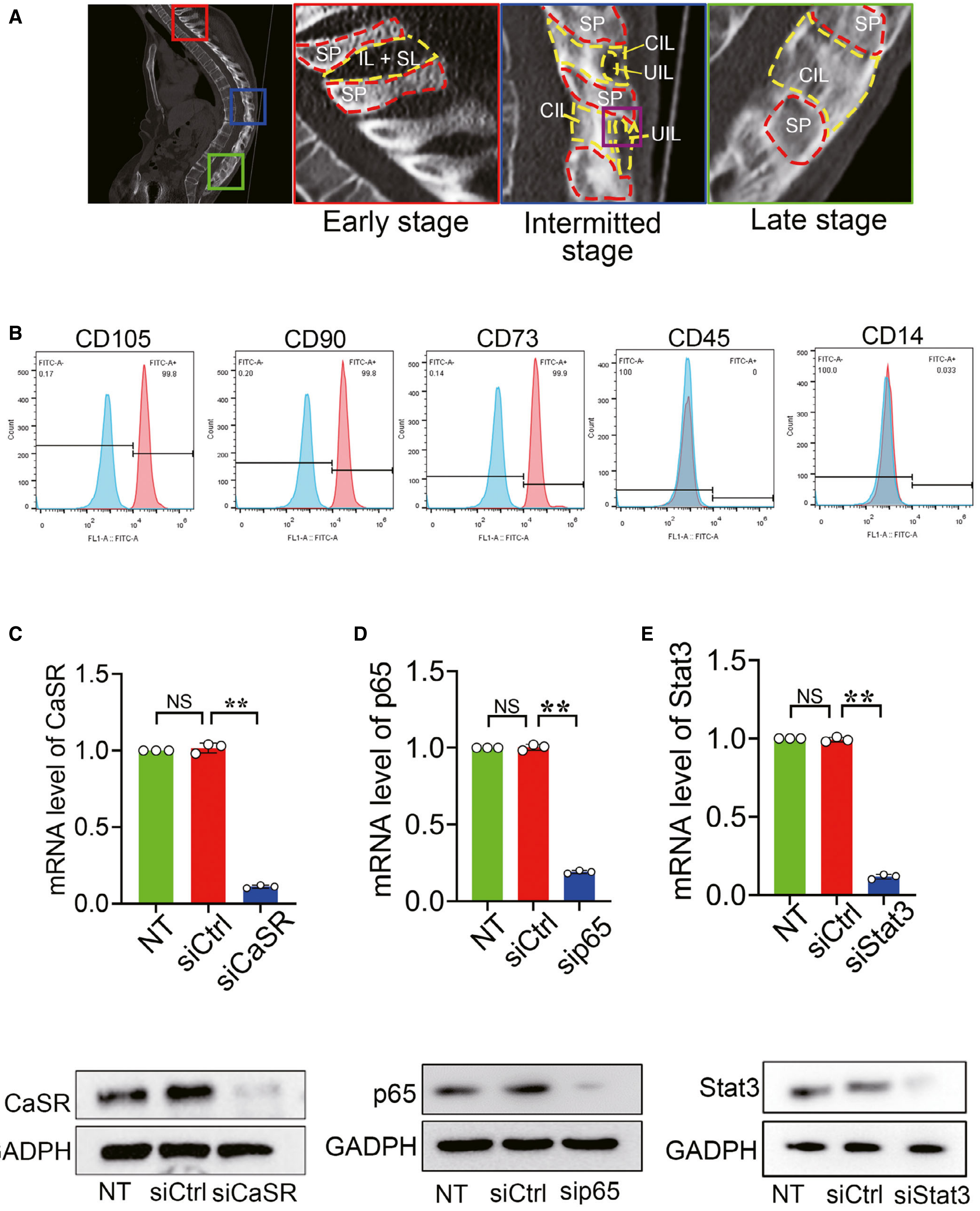

Figure EV5.

© 2020 The Authors
